# Beyond Local Footprints: Disentangling Large‐Scale Redistribution and Local Abundance Responses to Offshore Wind Farms

**DOI:** 10.1002/ece3.73256

**Published:** 2026-03-15

**Authors:** Moritz Mercker, Verena Peschko, Kai Borkenhagen, Nele Markones, Henriette Schwemmer, Volker Dierschke, Stefan Garthe

**Affiliations:** ^1^ Bionum GmbH, Consultants in Ecological Statistics Hamburg Germany; ^2^ Institute for Mathematics and Interdisciplinary Center of Scientific Computing (IWR) Heidelberg University Heidelberg Germany; ^3^ Research and Technology Centre (FTZ) University of Kiel Büsum Germany; ^4^ Federation of German Avifaunists (DDA) Münster Germany

## Abstract

A differentiated understanding of how regional human activities affect the spatial distribution and abundance of animals is of great ecological importance. However, estimating these effects from empirical data is challenging, as human activities influence animals in different ways and on various spatial and temporal scales. Additionally, spatio‐temporal animal abundance is often shaped by intrinsic and extrinsic factors, which can confound impact assessments. To separate these influences, we combined regression and mechanistic modelling. First, we used partial differential equations to simulate potential animal redistribution patterns driven by regional human activities. These patterns were then incorporated as predictors into regression‐based species distribution models, alongside other anthropogenic and environmental covariates. This allowed us to estimate and predict human‐induced changes by jointly accounting for pressure‐driven large‐scale redistribution and local changes in expected abundance, while controlling for additional environmental influences. We applied this approach to assess the impact of offshore wind farms (OWF) on common murres ( *Uria aalge* ) in the German North Sea during autumn. OWF constructed by 2019 reduced common murre numbers within German waters by 18.3%. If the planned OWF priority and reservation areas outlined in the German Marine Spatial Plan are implemented, the predicted net reduction within German waters would increase to 77.7%. Importantly, these predictions do not account for additional anthropogenic activities or OWF expansion in surrounding waters, which could further affect common murre abundance beyond the scenarios considered here. By comparing predicted animal numbers and distributions under hypothetical scenarios with and without specific human pressures, our method enables the quantification and prediction of human‐induced effects on regional trends and large‐scale redistribution. The framework provides a transparent way to disentangle large‐scale redistribution and cumulative effects from local responses in spatially bounded management areas, supporting impact assessments under ongoing expansion of offshore renewables.

## Graphical abstract

We combine regression‐based species distribution models with a mechanistic redistribution component to assess how regional human activities affect animal distribution and abundance across spatial scales. Applied to common murres ( *Uria aalge* ) in the German North Sea, the framework indicates scenario‐based reductions in numbers within German waters of 18.3% under the 2019 offshore wind farm configuration, increasing to 77.7% if currently planned areas are implemented. By explicitly distinguishing local avoidance from large‐scale redistribution, the approach supports transparent ecological impact assessments under phased development of renewable energy infrastructure.

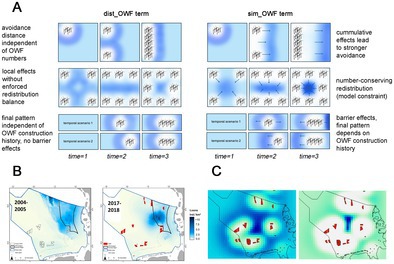

## 1 Introduction

**FIGURE 1:**
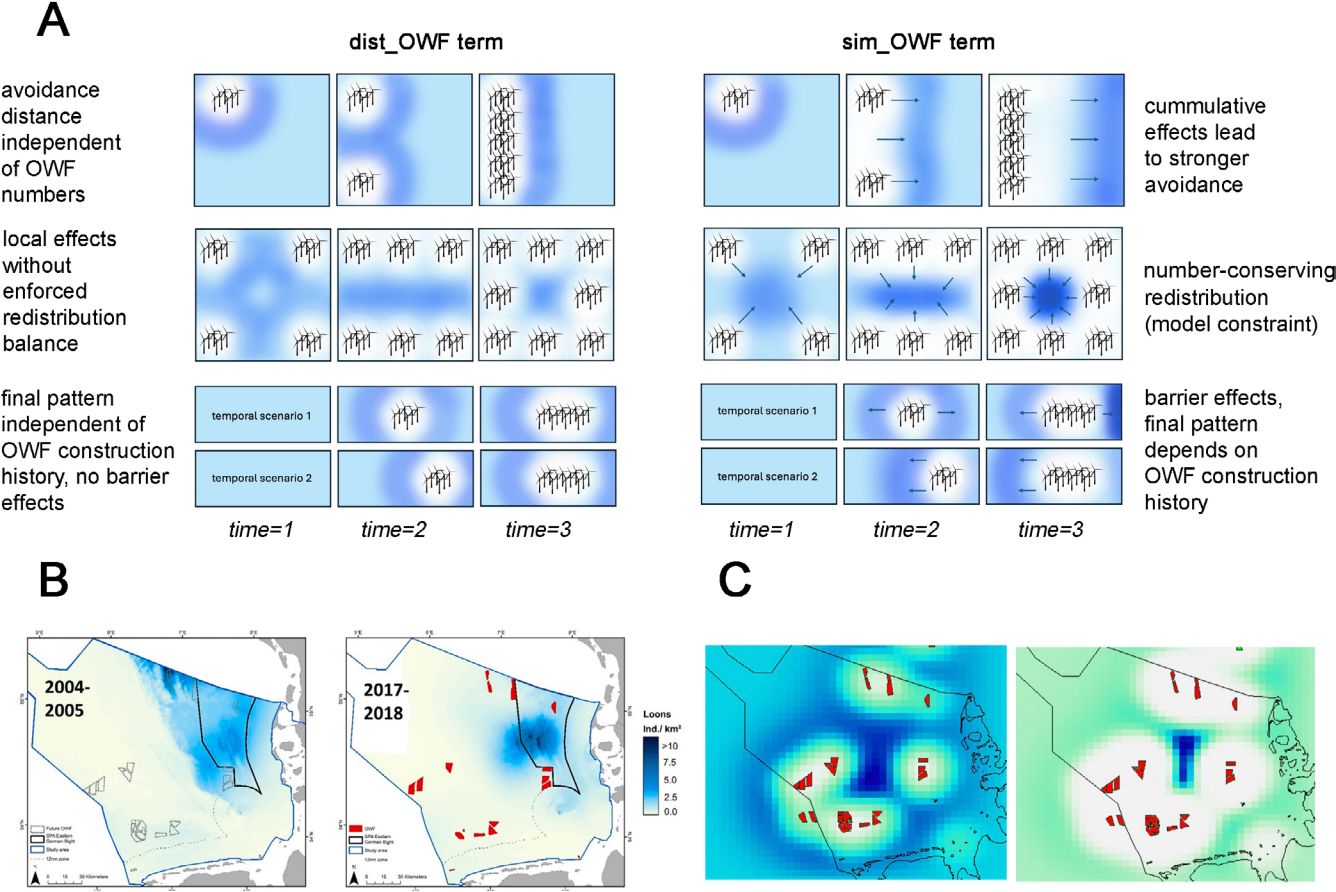
Conceptual schemes, empirical examples, and simulations illustrating why a distance‐only OWF covariate cannot capture cumulative and path‐dependent (i.e., dependence on development history) redistribution. (A) Conceptual comparison. The term dist_OWF (left‐hand side) represents local, static proximity effects to the nearest OWF (radial exposure), with final patterns determined solely by the final OWF configuration and without explicit fluxes, enforced redistribution balance, or path dependence. In contrast, sim_OWF (right‐hand side) represents redistribution driven by directional fluxes along an OWF‐induced stress gradient and number‐conserving movement, allowing cumulative effects of multiple OWFs to emerge as a model constraint. This formulation can generate barrier effects and path‐dependent final patterns that depend on the history of OWF construction. (B) Empirical example of large‐scale OWF‐associated distributional change that cannot be adequately described by simple or static distance effects (illustrative example using loons from Garthe et al. (2025), included to motivate the type of large‐scale displacement patterns captured by sim_OWF). (C) Example simulations of sim_OWF for different avoidance strengths (β), illustrating the resulting large‐scale redistribution patterns.

**FIGURE 2:**
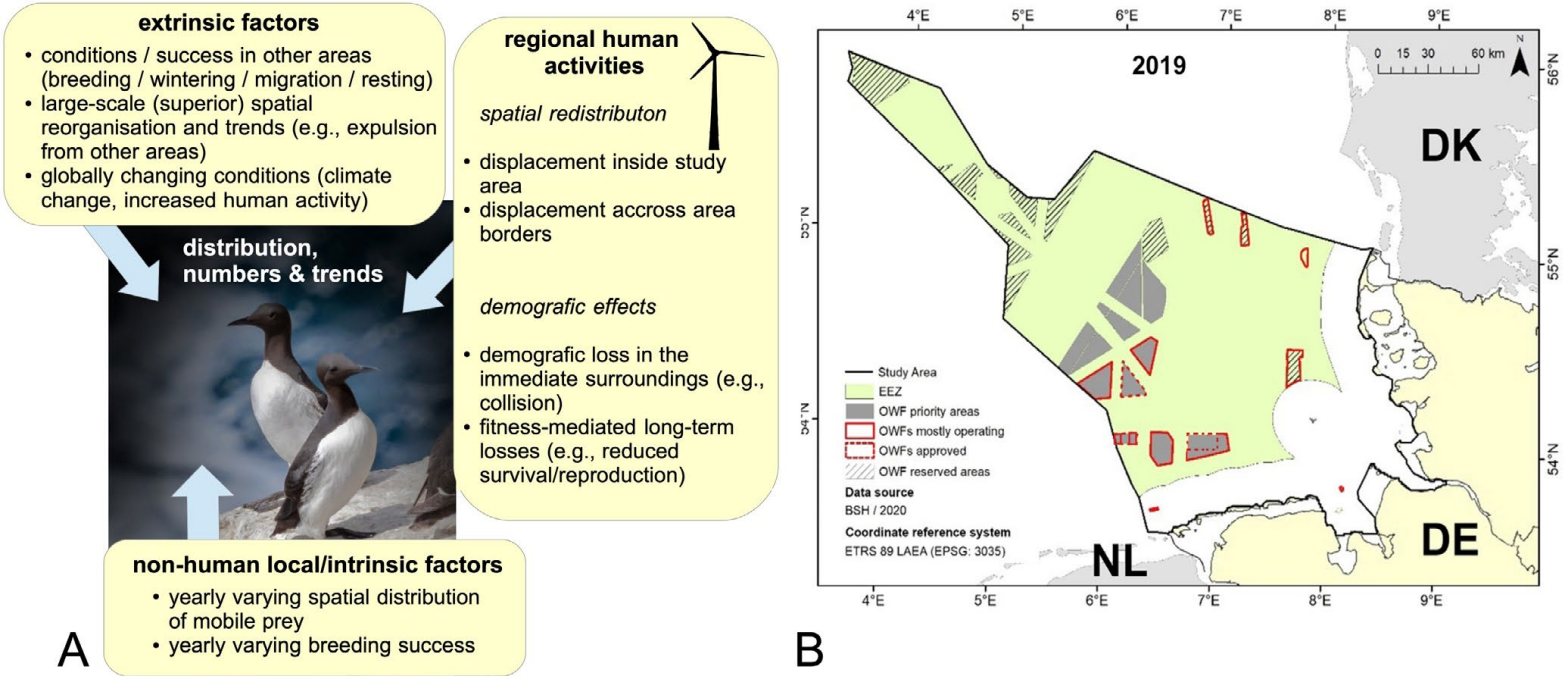
(A) Examples of factors intrinsic and extrinsic to the study area that could influence regional densities, spatial patterns, and trends in species such as common murres in the German North Sea. (B) Study area (German exclusive economic zone (EEZ) and coastal areas—black line) including operating OWF (red‐framed areas), planned (grey‐shaded areas) and optional/reserved (grey‐striped areas) for OWF construction. Figure modified from (BSH 2021).

Anthropogenic activities frequently lead to distinct changes in wildlife numbers and/or spatial patterns, and the robust quantification and prediction of these changes provides an important basis for ecosystem management (Lewis et al. 2021). Human activities can affect animals qualitatively in various ways and on different spatio‐temporal scales (Figures 1 and 2A). For example, animals within an area of interest might rearrange spatially (e.g., avoiding these activities on a local‐scale, in the offshore‐scenario considered in this study referring to a scale of kilometres), while their overall numbers may remain constant (Langridge et al. 2020). However, if suitable undisturbed habitat is limited or fragmented by the spatial overlap of multiple anthropogenic activities, redistribution may extend beyond the boundaries of the study area, displacing individuals on a large‐scale and effectively causing a loss from the focal region. Beyond these displacements, individuals may also be completely lost from the wider region, either by migrating to alternative distant regions or as a result of increased mortality. The latter may occur as a result of a direct reduction (e.g., by collision with anthropogenic structures Mercker and Jödicke 2021), or by secondary effects, usually over longer time scales (e.g., as a result of reduced prey availability; Carter et al. 2019 or increased stress; Tarjuelo et al. 2015, leading to reduced survival or reproduction; French et al. 2011).

In addition to specific human activities, several other factors intrinsic or extrinsic to the study area may influence species distribution and numbers, thus impeding impact analyses (Figure 2A). For example, animals can redistribute spatially within the study area depending on varying prey availability (Sveegaard et al. 2012), leading to high spatial variability and making it difficult to interpret the spatial distribution of animals within a given year. In addition, particularly in study designs where animal counts are conducted over extended spatial and temporal scales, the population is operationally open with respect to the study area, and yearly numbers within the considered area may also depend strongly on extrinsic conditions (e.g., in wintering or breeding areas), such as annual variations in reproductive success in breeding grounds, or stochastic or systematic large‐scale spatial variations. Overall, in study designs where populations are operationally open with respect to the area of interest, trend analyses may be an inappropriate tool for estimating the partial effect of regional human pressures on animal numbers within that area. This is because observed trends can be driven by multiple processes, including dispersal‐driven redistribution across space as well as demographic changes influenced by conditions external to the study area, which may strongly confound causal interpretation. For example, an externally driven positive population trend (e.g., caused by spatial displacement into the study area) could be locally damped by the simultaneous intensification of human activities within the area of interest, wrongly suggesting a lack of negative effects caused by these activities.

Two main (and distinctly different) theoretical tools have previously been developed and applied to investigate and model the influence of human pressures on spatio‐temporal animal distributions and density patterns, namely correlative/regression‐based (Elith and Leathwick 2009) and mechanistic (Kearney and Porter 2009; Evans et al. 2015) species distribution models.

Regression‐based methods are mainly data‐driven and correlate the observed species distribution with different covariates, such as environmental variables and/or those describing human activities. The advantage of these methods is that they generate results mainly driven by empirical data, without strong reliance on hypothetical mechanistic behavioural assumptions. In addition, regression methods can consider spatial or temporal patterns that are not explained by the considered covariables (i.e., phenomenological terms, such as trends), which is important for modelling realistic spatio‐temporal changes. A possible drawback of regression models, however, is that they provide information about correlations but not about causalities, which must be considered when interpreting the results. For example, a detected correlation between seabird abundance and fishing vessel density may indicate that the birds are attracted by the fishery process itself (e.g., due to discarding Schwemmer and Garthe 2005), or, in contrast, they could be disturbed by fishing vessels, but the birds and ships may both be attracted by a locally high fish abundance, which can lead to a spurious association between both variables if this shared driver is not accounted for (the ‘third‐variable problem’; Haig 1992). Furthermore, while regression models can describe associations between covariates and observed distributions, they are typically agnostic to the underlying movement processes that generate redistribution patterns over space and time. In particular, cumulative, number‐conserving redistribution driven by spatial pressure gradients and exhibiting path dependence (i.e., dependence on development history) can be difficult to represent using purely distance‐ or site‐based correlations alone without introducing additional structural assumptions. Finally, predictions based on regression approaches usually work well for parameter values within the experimentally assessed range (i.e., for interpolations), but predictions based on covariables outside the experimental range (i.e., extrapolations) are frequently connected to high uncertainties, and unrealistic results can be obtained.

In contrast, mechanistic modelling approaches (also called ‘process‐based models’) are strongly based on a priori (e.g., independently derived) information about species behaviour, and are thus based on specific mechanistic assumptions about the process of interest. Such approaches encode explicit hypotheses about how ecological processes operate and interact, while abstracting from fine‐scale variability that may be irrelevant for the question at hand. The advantage of these approaches is that these assumptions typically constrain the simulations such that predictions can be more robust under spatial or temporal extrapolation beyond observed conditions than purely regression‐based approaches, because mechanistic structure can limit the unbounded propagation of estimated relationships (Kearney and Porter 2009; Evans et al. 2015). However, this robustness depends on how well the underlying mechanisms are specified: if key processes or behavioural parameters are poorly known, misspecified, or not transferable across contexts, mechanistic predictions may become speculative or show systematic mismatches in predicted patterns (Evans et al. 2015; Yates et al. 2018). Partial differential equations (PDEs) are a class of mechanistic models that can describe various spatial ecological phenomena, and which are particularly suited to represent animal movement and redistribution as continuous movement processes (Holmes et al. 1994; Petrovski 2021). While partial differential equations are also used in a regression/correlative context—for example in stochastic PDE formulations employed by R‐INLA to represent spatial covariance structures (Zuur et al. 2017)—these applications serve a fundamentally different purpose, namely the approximation of spatial correlation (i.e., a latent random field) rather than the explicit representation of ecological state dynamics such as pressure‐driven redistribution. In contrast to individual‐based mechanistic models (e.g., Broder 2002; Stillman 2008), PDE‐based approaches are not limited by a maximal considered system size (e.g., a maximal number of interacting individuals due to computational constraints), and simulated processes operate at the population rather than the individual scale. This makes them well suited for large study areas, for parametrization based on large‐scale count data, and facilitates their integration with density‐based regression models, while retaining an explicit representation of large‐scale pressure‐driven redistribution processes. Despite these advantages, PDE‐based approaches remain comparatively uncommon in applied ecological impact assessments, in part because such redistribution‐based formulations require an explicit end‐to‐end specification and evaluation of dynamical components (e.g., flux terms, boundary conditions, temporal updating and numerical implementation), for which fewer standardised workflows exist than for purely correlative models.

A prime example for studying the impact of human activities on wild animal populations is provided by marine offshore habitats, where human activities (such as offshore windfarms (OWF)) are currently increasing, distinctly changing animal distributions and habitat properties (Mendel et al. 2019; Raul Vilela et al. 2020). Various modern regression‐based approaches have recently been presented and applied to estimate the effects of OWF on offshore seabird distributions and numbers (e.g., Mendel et al. 2019; Raul Vilela et al. 2020; Peschko, Mendel, et al. 2020; Mercker, Dierschke, et al. 2021; Peschko et al. 2021). These methods adequately describe local losses or gains in bird densities due to human pressures (e.g., in the 20 km surroundings of OWF) and can also detect regional trends caused by several interacting drivers. However, distance‐based approaches are inherently limited to describing local, static proximity effects and therefore do not explicitly represent large‐scale redistribution processes, in which individuals actively redistribute across space. Such large‐scale redistribution is intrinsically a movement process, characterised by directional fluxes, cumulative effects of multiple OWF, and potential path dependence of the final spatial pattern. Within the redistribution component of the model domain, local decreases in density imply compensatory increases elsewhere, whereas net changes at the scale of the focal area may still occur in open systems. In our framework, we therefore decompose OWF‐related change into (i) a local proximity term capturing local‐scale avoidance footprints and (ii) a mechanistic, redistribution‐based pressure‐driven term capturing large‐scale displacement patterns; importantly, empirical separability of these components depends on whether the data support distinct signal shapes for both predictors and on the assumed redistribution structure. As a consequence, impact assessments based solely on local OWF‐distance effects or on regional trends may provide an incomplete picture of OWF‐induced changes, because they do not account for large scale redistribution. This conceptual distinction between local exposure effects and large‐scale redistribution processes is illustrated in Figure 1. Indeed, previous OWF impact studies for similar species and regions (e.g., loons in German North Sea waters) estimated strongly comparable losses in the OWF surroundings and similar trends, but came to quite different conclusions (e.g., Raul Vilela et al. 2020; Garthe et al. 2023). One possible reason is that it was difficult or impossible to attribute observed regional changes in abundance or population unambiguously to OWF versus other large‐scale drivers. Thus it currently remains unclear how strong the regional loon population is affected by OWF despite strong local effects on distribution.

The above advantages and shortcomings of correlative and mechanistic approaches in the context of impact studies suggest the need to either apply both methods in a comparative manner, or alternatively to develop integrated (hybrid) methods to clarify and predict spatio‐temporal species distribution and population trends under changing conditions (e.g., Kearney and Porter 2009; Kearney et al. 2010; Dormann et al. 2012; Townsend Peterson et al. 2015; Rougier et al. 2015). In this study, we followed the latter strategy to improve the estimation and prediction of the changes in animal large‐scale distribution and numbers caused by regional human activities, by synergistically joining mechanistic and regression approaches. Notably, we extended previous regression approaches by making additional use of the predictive capacity of mechanistic PDEs, especially when measuring and predicting complex animal redistribution processes beyond simple correlations. We applied our approach to investigate the current and future impacts of intensified OWF implementation on common murres ( *Uria aalge* ) in the German North Sea. The analysis focuses on the species‐specific autumn period, which represents the phase with the highest abundances of common murres in the German North Sea. This period is also of particular ecological relevance, as murres undergo wing moult and are temporarily flightless, while still being in late stages of chick rearing. As a consequence, individuals may be particularly sensitive to additional anthropogenic pressures during this season, making it a critical window for assessing potential impacts of offshore wind farm development.

## 2 Materials and Methods

To facilitate scrutiny of the approach, we briefly summarise the analytical workflow before providing technical details. First, a mechanistic redistribution field (sim_OWF) was derived using PDE‐based simulations to represent large‐scale OWF‐induced displacement independent of local avoidance. Second, this redistribution component was integrated into a spatio‐temporal GAMM together with a distance‐based predictor (dist_OWF) capturing local‐scale effects, as well as environmental and temporal covariates. Third, the fitted model was used to generate scenario‐based predictions contrasting disturbed (with OWF) and hypothetical undisturbed conditions under otherwise identical background dynamics. Finally, these contrasts were used to quantify OWF‐induced changes in spatial distribution and net abundance within the German Exclusive Economic Zone (EEZ) and territorial waters, including uncertainty estimates. This stepwise structure ensures that each model component has a clear ecological interpretation and that scenario results can be understood without detailed knowledge of the underlying statistical formulation.

Throughout this study, we use the term population in an operational sense to denote the number of individuals of the focal species present within the defined management unit (German EEZ plus territorial waters in the German North Sea) during the analysed season (autumn) and survey time window. This operational population is explicitly open with respect to the focal area: changes in population size therefore refer to net changes in expected numbers within the area (i.e., redistribution across the boundary) rather than to demographic closure or mortality.

### 2.1 Study Area and Data

#### 2.1.1 Study Area

The study area considered as a case study is given by the German Exclusive Economic Zone and the German territorial waters in the German North Sea (total surface area: 41,334 km^2^; hereafter jointly referred to as ‘EEZ’ for simplicity) (Figure 2B), where several wind farms have been established within the last two decades (e.g., Figures 1B and 2B) with a capacity of 7.7 GW at the end of 2020. Current German Government plans include the implementation of at least 30 GW of offshore wind power in the German EEZ by 2030, at least 40 GW by 2035, and at least 70 GW by 2045 (The Federal German Government 2021; Deutscher Bundestag 2022). The current German marine spatial plan designates priority and reserved areas for OWF implementation as shown in Figure 2B. The draft of the site development plan, however, defines additional areas for OWF development to reach the implementation of 70 GW by 2045. Due to the ongoing planning process, we focused on the areas outlined in the marine spatial plan.

#### 2.1.2 Raw Bird‐Count Data and Data Processing

**FIGURE 3:**
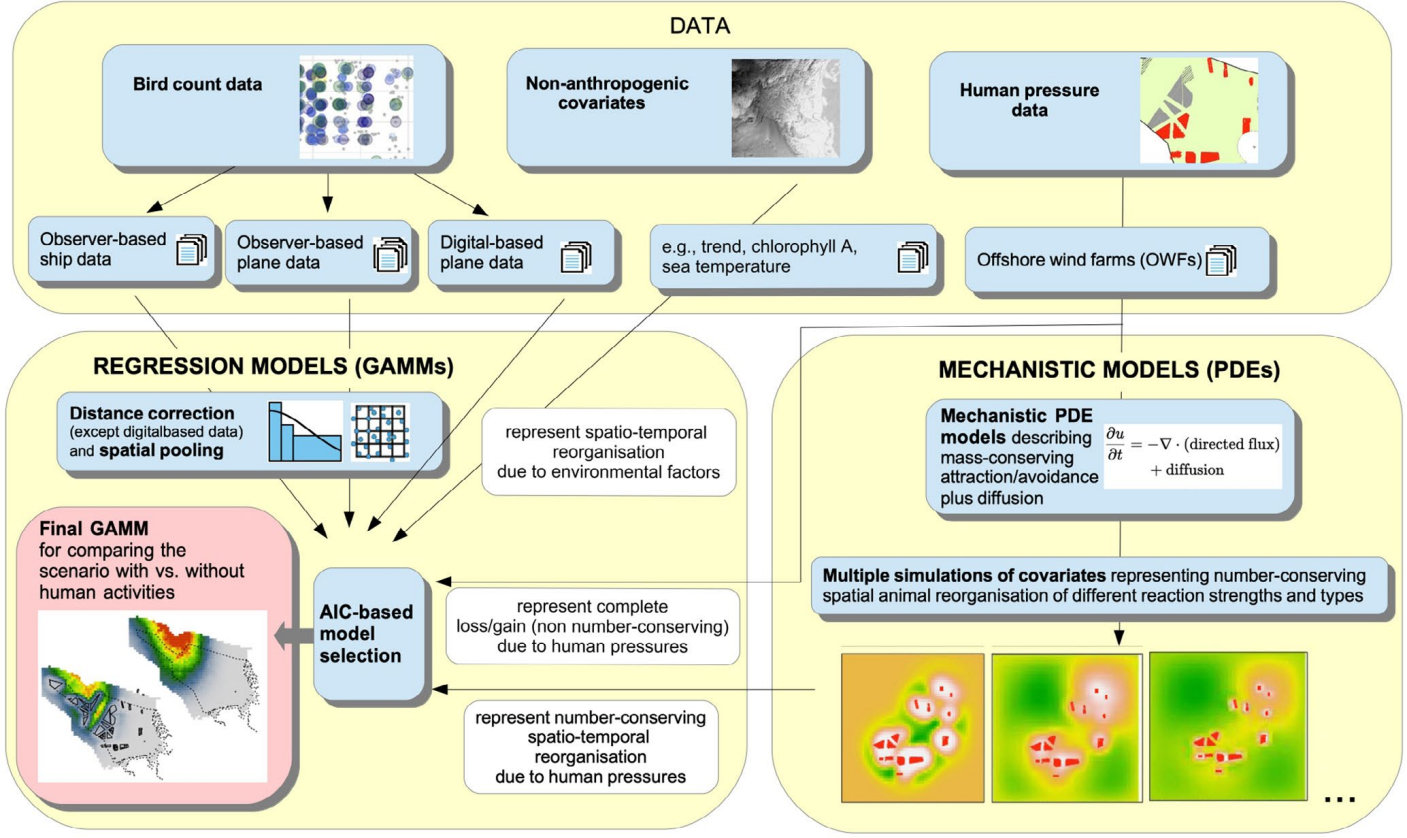
Technical overview of the present approach synergistically combining regression and mechanistic modelling to investigate and predict the effects of regional human activities on animal distribution and numbers within an area of interest. The approach has been applied to investigate the impact of intensified OWF activity in the German North Sea on the regional common murre population during autumn. The mechanistic component is illustrated schematically by a flux‐based partial differential equation representing number‐conserving spatial redistribution, in which u denotes a generic animal density state variable, $\partial \left(.\right)/\partial_{t}$ the time derivative, and $\nabla \cdot \left(\cdot \right)$ the mathematical divergence of directed movement fluxes; an additional diffusion term represents undirected movement.

A synopsis of the present approach is given in Figure 3. Several technical details, for example, with respect to imperfect animal detection during surveys, spatio‐temporal autocorrelation, data pooling and regression analyses are described briefly below, and a more detailed description can be found in (Mercker, Markones, et al. 2021). Raw bird‐count data were obtained from aerial (digital‐ and observer‐based) as well as vessel (observer‐based) surveys from 2003 to 2019, covering the main period of OWF construction within the German North Sea. Data were obtained from various seabird monitoring and research projects conducted by the University of Kiel (e.g., the German Marine Biodiversity Monitoring on behalf of the Federal Agency for Nature Conservation) and from environmental impact studies and monitoring during construction and operation of wind farms provided by the German Federal Maritime and Hydrographic Agency (BSH). Further details of the survey data are given in Table 1; further information on data sources and field methods can be found in for example, Mendel et al. (2019), Peschko, Mendel, et al. (2020), Garthe et al. (2023). The study was restricted to the species‐specific autumn period (16.07–30.09 Garthe et al. 2007), as the season with highest numbers of common murres in the German North Sea. Several technical details regarding data processing prior to the analysis (e.g., with respect to imperfect detection, spatio‐temporal autocorrelation, and data pooling) can be found in for example, Mendel et al. (2019), Peschko, Mendel, et al. (2020), Mercker, Dierschke, et al. (2021), Mercker, Markones, et al. (2021); in the following, only differences from these approaches are detailed.

**TABLE 1:** Summary of the survey data used in the analysis, including temporal coverage and survey platforms.

Metric	Value
Number of records (rows)	9918
Time coverage (years)	2003–2019
Number of unique survey dates	372
Season analysed	Autumn
Total (distance‐corrected) bird count sum	69,838
Proportion zeros	70.1%
Total surveyed area	44,356 km^2^
Records by platform	1 = 3753 (vessel observer)
	2 = 1645 (aerial digital)
	3 = 4520 (aerial observer)

Distance‐corrected bird‐count data, locally surveyed area sizes, and covariate values were spatio‐temporally pooled using a regular 8 × 8 km spatial grid, depicting the optimal compromise between a manageable autocorrelation while keeping a sufficient spatial resolution (Mercker, Dierschke, et al. 2021; Mercker, Markones, et al. 2021). In particular, bird‐count data and monitored areas were summed separately for each unique combination of grid cell‐ID, year and season, whereas all other covariates (including spatial coordinates) were averaged. This led to a much higher effective spatial resolution than a 8 × 8 km if grid‐cell centres had been used instead (Mercker, Markones, et al. 2021). Spatial plots of the pooled common murre bird‐count data are given in Figure S1A–B. Final pooled data (without data from OWF construction phases—cf., below) comprised 9918 data points with 30,186 observed (resulting in 69,838 distance‐corrected) individuals based on 44,356 monitored km^2^. In particular, 18,480 individuals were counted before construction of the OWF, whereas 11,706 individuals were counted in phases where OWF were operating (cf., Table 1).

#### 2.1.3 Data on Human Activities

Data on wind turbine locations from 2003 to 2019 were obtained from the OSPAR Data and Information Management System (link), and construction‐ and operation‐phase dates for several OWFs were acquired from various online sources. In addition to OWFs within the German EEZ, those close to but outside the EEZ boundary (namely those belonging to the Danish Horns Rev. complex and the Netherlands Gemini parks) were also considered. Planned OWF areas (priority and reserved areas) for future implementation were obtained from the marine spatial plan for the German EEZ 2021 (link). Before merging pooled bird‐count data with spatio‐temporal OWF information, we removed data from OWF‐dependent construction phases to reduce the complexity of the analysis. Subsequently, the distance to the nearest operating OWF was assigned for each (pooled) spatio‐temporal observation (with respect to the time point of the observation), with distances > 20 km truncated at 20 km. This truncation is based on published evidence suggesting that local‐scale displacement responses of seabirds to OWFs are typically strongest within the first tens of kilometres and decline with distance (Mendel et al. 2019; Raul Vilela et al. 2020; Peschko, Mendel, et al. 2020; Peschko et al. 2024). Accordingly, the distance‐based component dist_OWF is formulated under the modelling assumption that any remaining local‐scale influence beyond 20 km is limited. However, we acknowledge that such effects cannot be fully ruled out; if present, they would not be captured by the truncated local distance term and would constitute a source of residual uncertainty in the analysis.

#### 2.1.4 Environmental Covariates

We also considered the effect of natural covariates on birds by investigating different variables potentially representing prey availability and/or suitable habitats. Annual spatial chlorophyll A patterns were obtained from the OceanColor Web provided by National Aeronautics and Space Administration (NASA) (link), as a proxy for annually varying primary production. In addition, spatially varying sea surface temperature was obtained from the NASA Moderate Resolution Imaging Spectroradiometer database (link). To also detect and describe oceanographic fronts, which are frequently sites of enhanced physical and biological activity potentially attractive for seabirds (Schneider 1990), we also calculated spatial gradients of sea surface temperature as covariates (which is however only possible for rather static fronts). The nearest distance to the coastline was obtained from NASA Ocean Colour Web (link) and water depth (gridded bathymetry data) was obtained from General Bathymetric Chart of the Oceans (GEBCO) 2020 Grid (doi:10.5285/a29c5465‐b138‐234d‐e053‐6c86abc040b9) (link).

After careful evaluation, we excluded the additional variables ‘water depth’ and ‘nearest distance to the coast’ from the spatial impact analysis (but not from the trend analysis; cf. below). Both variables are spatially associated with OWF locations, and their inclusion in the spatial impact model would therefore introduce strong collinearity, inflating standard errors and complicating the interpretation of OWF‐related effects. In principle, such confounding can be addressed using a classical before–after control–impact (BACI) design, which contrasts temporal changes in impacted areas against stable control areas (e.g., Mendel et al. 2019; Peschko, Mendel, et al. 2020; Garthe et al. 2023; Peschko et al. 2024). However, a strict BACI design is difficult to apply in the present modelling context. In particular, OWF development in the German North Sea has occurred in a spatially and temporally staggered manner, which precludes the definition of a single, well‐defined ‘before’ and ‘after’ period that could be applied consistently across the entire study area. In the current study, we aim to describe time‐dependent, large‐scale bird redistribution at the scale of the entire system, which requires a temporally coherent scenario definition rather than multiple park‐specific before–after contrasts. Importantly, this does not imply that BACI‐based analyses are generally unsuitable for the German North Sea. On the contrary, BACI designs have been successfully applied in this region to quantify local effects, as well as in large‐scale cumulative assessments (e.g., Garthe et al. 2023). Rather, the limitation arises in the context of the present modelling approach, which aims to represent spatially continuous and temporally dynamic redistribution processes at the system scale. Moreover, the proposed modelling framework is designed to be transferable across multiple anthropogenic stressors (e.g., shipping activity), some of which cannot be meaningfully analysed within a classical BACI framework. However, because OWF locations are not randomly distributed in space, local OWF proximity can be correlated with underlying habitat gradients. Consequently, apparent OWF‐proximity effects may partly reflect baseline differences in bird density along gradients such as water depth or distance to the coast (confounding by spatial co‐location). As described below, we therefore approximate and integrate BACI‐like contrast information at later stages of the analysis to mitigate this confounding risk, without imposing a strict before–after control–impact design. To further account for spatially inhomogeneous bird distribution patterns not explained by the included predictors, we additionally incorporated spatial coordinates as smooth two‐dimensional predictors, restricting the maximum number of knots to nine to avoid collinearity between the spatial smooth and OWF locations.

### 2.2 Mechanistic PDE Models and Simulations

Our mechanistic model of animal redistribution is based on the Keller‐Segel model, originally developed to describe the interplay between directed chemotaxis and random (diffusive) movement of biological units (such as bacteria) (Keller and Segel 1971). Here, the density $n\left(t;x\right)$ of cells and the chemoattractant concentration $c\left(t;x\right)$ were considered, where t represents the time and x a 2D space variable. The interplay of chemotaxis in combination with the production of the chemoattractant is given by the following system of two coupled PDEs, namely:

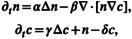

for t>0, $x\in {\mathbb{R}}^{2}$ and appropriate initial and boundary conditions. Here, $\partial_{t}$ depicts the time derivative, Δ the Laplace‐operator, ∇ the spatial gradient and ∇⋅ the divergence operator. In the first equation, Δ describes diffusive/random (isotrop) movement behaviour scaled by the factor α, whereas the term $\nabla \cdot \left[n\nabla c\right]$ describes the directed attraction movement along the gradient of c. The second equation describes the secretion of c by the biological units n, including diffusion and degradation.

We adapted this model to describe animal displacement induced by regional human activities. First, in our model, $n\left(\vec{x};y\right)$ describes the density of animals (with year y and spatial location $\vec{x}=\left(\mathrm{lon};\mathrm{lat}\right)$). The variable $c\left(\vec{x};y\right)$ represents the spatial impact field of a human pressure of interest (cf. below), whereas the location of the latter is given by the variable $\zeta \left(\vec{x};y\right)$. In our exemplarily application, $\zeta \left(\vec{x};y\right)$ describes the location of OWF as a binary variable (inside vs. outside the OWF). The variable ζ is not used directly, because the impact of anthropogenic structures is typically not confined to their exact footprint, but extends into the surrounding area due to attraction or avoidance responses mediated, for example, by visual perception, spatial memory (Mercker, Schwemmer, et al. 2021), sound or vibration (Popper et al. 2022). Indeed, for OWF, seabird responses have been documented over distances reaching or even exceeding 20 km (Mendel et al. 2019; Raul Vilela et al. 2020; Garthe et al. 2023; Peschko et al. 2024). To construct a spatially continuous pressure field from the binary OWF mask ζ, we applied a diffusion‐based smoothing step. Specifically, we considered the solution c of the diffusion equation $\partial_{t}c=\gamma \Delta c$ with initial condition $c\left(\vec{x};t=0\right)=\zeta \left(\vec{x};y\right)$, evaluated separately for each year y after a small number of numerical time steps. This procedure is mathematically equivalent to an isotropic Gaussian smoothing of ζ, with γ controlling the effective spatial scale of influence. Importantly, the purpose of this step is not to model animal movement mechanistically, but to derive a smooth, scale‐controlled pressure field that accounts for both distance to OWF and the spatial extent of OWF clusters. In contrast to simple distance‐decay functions centred on OWF boundaries, this formulation naturally yields stronger and more extensive pressure fields for larger OWF areas, while small or fragmented OWF patches are smoothed out more rapidly.

The resulting spatio‐temporal variable c is subsequently rescaled to the range [0,1] across all considered years y. This rescaling serves a purely technical purpose: it yields a dimensionless pressure field with a comparable numerical range across years and scenarios, thereby improving numerical stability and interpretability when c is later used as an input to the redistribution simulations and as a predictor in regression‐based models. Importantly, this step does not alter the relative spatial structure of the pressure field, which is the quantity of interest, but merely fixes its scale. Alternative linear rescalings would be equivalent and would not affect the qualitative behaviour of the simulations or the inferred effects. $c\left(\vec{x};y\right)$ hence represents the spatio‐temporally varying human pressure $\zeta \left(\vec{x};y\right)$, augmented by an isotropic long‐range effect with strength γ. In particular, for γ=0, c coincides with ζ, whereas for increasing γ, c spreads progressively from the original source of human activity. Examples of spatial patterns of c for different values of γ are given in Figure S2, considering operating OWF in German North Sea waters in 2019.

To derive a process‐informed, large‐scale redistribution index for use as a predictor in regression‐based analyses, we combined two conceptually distinct but computationally simple PDE steps: (i) construction of a smooth spatial pressure field from OWF geometry (as described above) and (ii) transport‐based propagation of animal densities in response to this pressure, formulated without intrinsic source or sink terms (i.e., conservative redistribution in the interior). Thus, for each year y separately, we first considered (and numerically solved) the following PDE describing the human‐induced pressure by:

(1) $$\partial_{t}c=\gamma \Delta c\;\text{with}\;c\left(\vec{x};t=0\right)=\zeta \left(\vec{x};y\right),$$

and subsequently the PDE simulating animal redistribution by:

(2) $$\partial_{t}n=\alpha \Delta n-\beta \nabla \cdot \left[n\nabla c\right],$$

both for a defined number of time steps δt (cf. below). Here, $\partial_{t}$ is the time derivative with respect to the numerical time variable t, γ scales the spatial reach of the impact of the human activity, α quantifies the amount of undirected (random‐like) animal movement, and β scales the strength of avoidance or attraction. The parameter ranges used for γ, α, and β in the simulation experiments are specified in the following section. Examples of abundance patterns resulting from different strengths of β are given in Figure S2. Numerical simulations after eight numerical time steps δt were considered separately for each year. As initial conditions in our simulations, for $n\left(\vec{x};y\right)$, we used the constant value of $n\left(\vec{x};y_{\text{start}}\right)=1$ for the first considered year 2003, and the respective foregoing solution $n\left(\vec{x};y-1\right)$ for all following years. Thus, if human activities changed in time, the stepwise spatial redistribution of animals due to the changing pressure locations and strengths was simulated. Redistribution simulations were conducted on a regular, rectangular spatial domain (EEZ plus buffer—cf., Figure S3A) and are therefore independent of the geometric shape of the German EEZ, which is applied only in post‐processing. We used Dirichlet boundary conditions with constant abundance $n\left({\vec{x}}_{\text{bound}};y\right)=1$ at the outer boundary, representing unchanged background density. The domain extent (longitude 3.0°–9.0°, latitude 53.0°–56.5°) was chosen such that the outer boundary is either close to the coastline or sufficiently distant from the German EEZ boundary to minimise any influence on redistribution patterns within the EEZ; boundary effects on EEZ‐level results are expected to be negligible. This two‐step PDE procedure is not intended as a detailed mechanistic population model. Instead, it generates a smooth, spatially and temporally consistent redistribution field that captures cumulative, large‐scale displacement patterns induced by OWF, while aiming for effectively preserved total abundance in the interior (cf. calibration step below). The resulting variable $n\left(\vec{x};y\right)$ is interpreted as a relative redistribution index rather than an absolute density prediction.

A regular spatial grid with 12,100 grid cells was used during PDE simulations. The final annual, spatially explicit field n (proportional to relative animal density as obtained from the redistribution simulations) was log‐transformed prior to entering the regression model to ensure consistency with the log‐link function used in the regression‐based analyses (cf. below). The resulting values were subsequently rescaled to the range [0,1] across all considered years y to improve numerical stability and convergence during model fitting. The resulting variable sim_OWF therefore represents a dimensionless, log‐density‐based redistribution field that captures relative large‐scale spatial redistribution with respect to OWF locations and is used as a spatio‐temporal predictor in the regression models (cf. below).

In summary, the variable sim_OWF is proportional to animal density and represents spatial large‐scale redistribution (especially active, time‐dependent movements in space—cf. Figure 1) of the birds in response to the implementation of the OWF. It is given by the solution of differential equations originally developed to describe chemotaxis and can be used in the following as an additional predictor in regression‐based species distribution modelling. The redistribution simulated by sim_OWF is constructed to be number‐conserving within the mechanistic model domain (German EEZ plus a spatial buffer), meaning that individuals are redistributed spatially but not created or removed by the transport process itself. This assumption does not imply a spatially closed or demographically closed population for the German EEZ. Instead, it represents a modelling constraint that isolates large‐scale redistribution from changes in overall abundance, which are estimated separately in the statistical model (cf. below). By allowing individuals to move out of the EEZ into the surrounding buffer, this approach enables the quantification of net changes within the EEZ under OWF scenarios while assuming, conservatively, that adjacent areas are available as evasive space (although OWF development in neighbouring waters may further limit such space in reality). In this sense, the number‐conserving constraint applies only to the mechanistic redistribution component and serves to distinguish large‐scale spatial redistribution from local losses or gains.

### 2.3 Integrative Generalised Additive Mixed Model (GAMM)‐Based Regression Modelling

The scheme for synergistically joining PDE‐ and regression‐based methods is shown in Figure 3 and is given by a two‐step procedure. In the first step, several thousand different possible anthropogenically induced redistribution patterns were simulated across multiple simulation rounds based on mechanistic PDE models (cf., above) and subsequently used as potential predictors in regression GAMM‐based species distribution models, including the simultaneous consideration of additional (natural and/or phenomenological) predictors. In the second step, model‐selection procedures were applied to select the simulated pattern that best explained the empirical animal redistribution data, eventually leading to the final regression model used for the impact analysis.

The detailed scheme is described in the following. First, 1000 different PDE‐based simulations of animal redistribution (Equations 1 and 2) were performed, where each simulation used stochastic values for $\gamma \in \left[0,100.0\right]$, $\alpha \in \left[0,0.1\right]$ and $\beta \in \left[0,100.0\right]$ (for an explanation of these parameters, see the derivation of the mechanistic formulas and/or Figure S2). Reasonable ranges for these parameters were determined heuristically. GAMMs (Hastie and Tibshirani 1990; Wood 2017; Zuur et al. 2009) were applied separately for each run, using the corresponding simulated pattern (variable sim_OWF—cf., above) as predictor, along with other investigated covariates. In particular, the most comprehensive GAMM was given by:

(3) $$\begin{array}{l} \eta_{\mathrm{ij}}=\beta_{0}+\beta_{i}+\text{metho}d_{j}+\mathrm{sea}\_\text{stat}e_{j}+s\left(\mathrm{yea}r_{j}\right)\\ \;\;+s\left(\mathrm{chl}A_{j}\right)+s\left(\text{sstem}p_{j}\right)+s\left(\mathrm{dx}\_\text{sstem}p_{j}\right)\\ \;\;+\mathrm{te}\left(\text{latitud}e_{j},\text{longitud}e_{j}\right)\\ \;\;+s\left(\text{dist}\_\mathrm{OW}F_{j}\right)+\mathrm{sim}\_\mathrm{OW}F_{j}\\ \;\;+\mathrm{la}g_{1,j}+\mathrm{la}g_{2,j}+\mathrm{la}g_{3,j}\\ \;\;+\text{offset}\left(\log\left(\mathrm{are}a_{j}\right)\right). \end{array}$$

The response $y_{\mathrm{ij}}$ denotes the (pooled, distance‐corrected) bird counts within a spatial sampling unit (an 8 × 8 km grid cell), where the index j refers to the sampling unit (grid cell) and the index i to the random intercept representing intra‐seasonal changes by defining intervals of 10 days. The model is fitted with a log‐link function such that $\log\left(\mu_{\mathrm{ij}}\right)=\eta_{\mathrm{ij}}$, where $\mu_{\mathrm{ij}}=E\left[y_{\mathrm{ij}}\right]$. Conditional on $\mu_{\mathrm{ij}}$, the counts are assumed to follow either a negative binomial distribution (Andreas and Samu 2011) or a Tweedie distribution (Kokonendji et al. 2004) (selected as described below). The random intercepts are assumed to be normally and independently distributed, $\beta_{i}∼N\left(0,\sigma_{2}^{2}\right)$. Here, $\beta_{0}$ is the fixed intercept, $s\left(.\right)$ depicts a smooth regression spline, and $\mathrm{te}\left(.\right)$ a tensor product 2D spline (Wood 2017). ${\text{method}}_{j}$ and $\mathrm{sea}\_{\text{state}}_{j}$ are related to the observation platform and wind conditions, respectively, both influencing the distance‐independent detectability (both variables are however also considered in an additional distance‐dependent detection correction step Mercker, Markones, et al. 2021). $s\left({\text{year}}_{j}\right)$ represents a (possibly nonlinear) temporal trend, frequently influenced by various factors intrinsic and extrinsic to the study area (cf. above), and $s\left({\text{chlA}}_{j}\right)$ represents the (possibly nonlinear) dependency on chlorophyll A concentration. ${\text{sstemp}}_{j}$ is the sea surface temperature and $\mathrm{dx}\_{\text{sstemp}}_{j}$ is its spatial gradient. ${\text{latitude}}_{j}$ and ${\text{longitude}}_{j}$ are spatial coordinates, and the corresponding two‐dimensional smooth is restricted to a limited number of degrees of freedom, as described above, to avoid collinearity. To account for small‐scale spatio‐temporal autocorrelation in the pooled sampling‐unit time series, we include three lag predictors (${\mathrm{lag}}_{1,j}$–${\mathrm{lag}}_{3,j}$) on the linear predictor scale. Here, ${\mathrm{lag}}_{1,j}$, ${\mathrm{lag}}_{2,j}$, and ${\mathrm{lag}}_{3,j}$ represent a Markov‐type dependence on the first, second, and third preceding pooled sampling units in the observation sequence, with an exponential down‐weighting by the elapsed time between observations. This time‐dependent decay ensures that preceding observations with large time gaps (e.g., from different surveys) contribute negligibly, while short‐range dependence between closely spaced observations is captured. This predictor‐based lag formulation differs technically from residual‐scale autoregressive correlation structures; however, under equidistant time steps it yields very similar results to an AR(3)‐type specification, as demonstrated by direct comparison in (Mercker, Markones, et al. 2021).

The two OWF‐related predictors $\text{dist}\_{\mathrm{OWF}}_{j}$ and $\mathrm{sim}\_{\mathrm{OWF}}_{j}$ (hereafter for simplicity dist_OWF and sim_OWF) represent fundamentally different model classes and are therefore not interchangeable. The distance‐based term dist_OWF describes a static, local exposure effect as a function of the minimum distance to the nearest offshore wind farm. It is purely scalar (radially symmetric) and does not encode directional fluxes or any coupling between local decreases and compensatory increases in animal density. Consequently, dist_OWF cannot represent cumulative effects of multiple OWF, does not enforce any redistribution balance, and yields final patterns that depend only on the final OWF configuration (no path dependence). In contrast, the simulated term sim_OWF represents a dynamic, large‐scale redistribution process derived from a mechanistic transport model. It explicitly represents directional fluxes along an OWF‐induced stress gradient and is constructed to be approximately number‐conserving, such that local decreases imply compensatory increases elsewhere. As a consequence, multiple OWF can interact cumulatively and the final pattern can be path‐dependent, reflecting barrier effects and construction history. This conceptual distinction is illustrated schematically in Figure 1.

Notably, both OWF‐related variables might also reflect positive/attraction effects, in which case, a negative correlation with these variables would be estimated during regression. Because survey effort varied per sampling unit, the logarithm of the locally surveyed area was included as an offset (Korner‐Nievergelt et al. 2015). The appropriate probability distribution with respect to the subsequently applied GAMM was first selected for each of the 2200 PDE‐based simulated patterns separately (based on the Akaike information criterion (AIC) Akaike 1973) from the negative binomial (Andreas and Samu 2011) and the Tweedie distribution (Kokonendji et al. 2004), using the above presented most comprehensive model. This distribution choice was used only for the respective intermediate model fits at this stage; it was not used to constrain the subsequent averaging of PDE parameters (see below). In addition, although a large number of model variants were fitted, this does not represent an unstructured or exploratory model search. All models share the same core structure, covariate set and biological hypotheses, and differ only in the parameterisation of a common redistribution framework (e.g., strength and direction of avoidance versus attraction, and relative contributions of diffusive versus directed movement). Model comparison using AIC was therefore applied to compare closely related variants within this constrained hypothesis space, rather than to select among qualitatively different model formulations. Conceptually, this procedure is analogous to a structured, grid‐based calibration of a fixed PDE model class, in which alternative parameterisations of the same redistribution process are evaluated to identify regions of parameter space that best approximate the observed spatio‐temporal patterns.

Next, for each simulated PDE‐based redistribution pattern, an AIC‐based predictor selection was applied, testing 42 biologically reasonable subsets of the predictors $s\left({\text{chlA}}_{j}\right)$, $s\left({\text{sstemp}}_{j}\right)$, $s\left(\mathrm{dx}\_{\text{sstemp}}_{j}\right)$, $s\left(\text{dist}\_{\mathrm{OWF}}_{j}\right)$ and $\mathrm{sim}\_{\mathrm{OWF}}_{j}$, where $\log\left(1+\text{dist}\_{\mathrm{OWF}}_{j}\right)$ instead of $\text{dist}\_{\mathrm{OWF}}_{j}$ was also tested to better describe local‐scale effects / ‘footprints’ (Mercker, Dierschke, et al. 2021). The model with the lowest AIC value was finally selected. To exclude possible unrealistic models per se, models in which both OWF‐related variables were contradictory (i.e., one variable suggested an attraction while the other measured a disturbance) were excluded; however, this was rarely the case in practice.

This finally led to 2200 different AIC‐values (related to the different simulated PDE‐based patterns), importantly including those models in which OWF‐related variables were not (or only partially) selected. At this stage, AIC values were used to characterise the relative support of the different PDE‐based redistribution patterns, rather than to identify a single globally optimal solution. In a second step, the entire simulation procedure was repeated 350 times, narrowing γ‐, α‐ and β‐parameter ranges to those associated with the models showing the 100 lowest AIC values from the first run. Thus, the first run evaluated the entire reasonable PDE parameter space, whereas the second run was restricted to the region where models showed low AIC values, thus locally refining the global AIC minimum, the latter representing the most appropriate simulated PDE‐based pattern describing OWF‐induced spatial animal redistribution.

In the last step, the final species distribution model (termed ‘sdGAMM’) was defined and fitted and subsequently used to analyse the current and future impacts of selected human activities (cf., below). Here, to increase the robustness of the PDE‐based results (respectively to minimise the selection/influence of random correlations due to the high number of tested predictors), average PDE parameters from the six models with the lowest AIC values and comprising the predictor sim_OWF were used to generate the final variable sim_OWF based on the PDE scheme described above. For this averaging step, we intentionally ignored the probability distribution selected for the individual intermediate model fits, because the averaging targets the mechanistic PDE parameterisation underlying the simulated redistribution field rather than the statistical error model. Using this final variable, predictor selection (cf., above) was again applied to obtain the final sdGAMM. Model validation was done using various residual‐based plots, for example, as described in (Korner‐Nievergelt et al. 2015; Zuur and Ieno 2016). The final probability distribution for the final sdGAMM (based on the averaged‐parameter sim_OWF) was selected by AIC, analogously to the procedure described above.

For the estimation of (possibly nonlinear) temporal trends resulting from multiple drivers (including OWF‐induced changes as well as other external and intrinsic factors), we modified the sdGAMM defined above. In particular, we used a two‐step modelling approach. Our objective was to separate (i) the overall temporal trend under the observed scenario with OWF from (ii) a counterfactual trend that approximates system dynamics in the absence of OWF. To estimate the temporal trend under the observed (real) scenario, we fitted a modified version of the sdGAMM in which all OWF‐related predictors were removed. In this trend model, we added smooth terms for water depth and nearest distance to the coast as predictors. These variables were included to represent broad‐scale, static habitat structure because OWF locations are spatially associated with environmental gradients such as water depth and distance to the coast, and removing OWF‐related predictors alone does not remove this spatial association. By conditioning the trend estimate on these habitat gradients, we reduce the risk that spatial habitat preferences are absorbed into the temporal trend component. The resulting trend therefore represents the best estimate of temporal change conditional on broad‐scale habitat structure under the observed OWF scenario. To approximate a counterfactual trend in the absence of OWF, we then combined two components: (i) the temporal trend estimated from the modified model without OWF‐related predictors, and (ii) the OWF‐induced annual percentage change estimated independently from the full sdGAMM that included the OWF‐related terms. In this way, the counterfactual ‘no‐OWF’ trend is obtained by adjusting the observed‐scenario trend by removing the OWF‐related annual change component inferred from the full model.

### 2.4 Population Estimates, Predictions, Robustness and Uncertainties

The geographical square used as the virtual area for PDE simulations (cf., above) was used as a basis for model‐based predictions. This area was further restricted to the German EEZ augmented by a regular belt of 40 km such that the total considered area (concerning water‐covered areas) roughly corresponded to twice the area of the German EEZ. The rationale behind this restriction was that the belt lying outside the study area contained sufficient area to incorporate displaced birds, while the geographic extrapolation was distinctly limited, preventing unrealistic predictions (e.g., with respect to the estimated preference of environmental covariates). Thus, the expression number‐conserving in this work concerns the region including the EEZ as well as the above‐mentioned belt. However, assuming that this belt was an undisturbed area that might take up displaced birds was only true to a very limited extent when considering future scenarios, due to OWF plans by neighbouring countries not included in this study.

**FIGURE 4:**
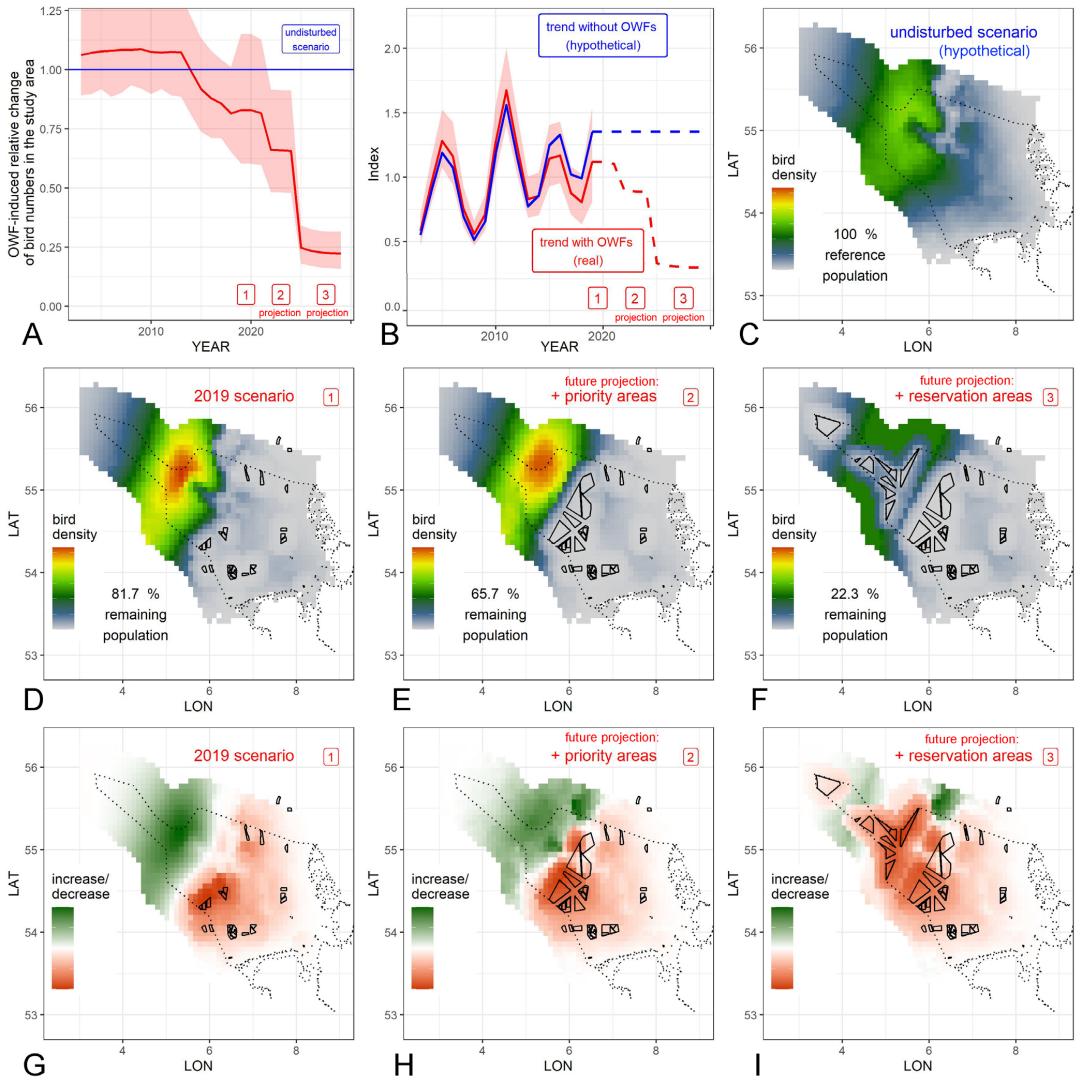
Predicted changes in numbers and distribution patterns of common murres during autumn in the study area (German EEZ and coastal waters) and surroundings. (A) Relative changes in bird numbers within the study area (solid red line) due to OWF compared with predicted number without OWF (blue line). Red‐shaded areas: 95% confidence bands. (B) Nonlinear trend estimates (trend index rescaled to a mean of 1.0) including the effect of existing (solid red line) and planned (dashed red line) OWF. No OWF‐independent trend is assumed from 2019 onwards, due to difficulties of extrapolations. Blue line: Analogous but hypothetical scenario without OWF. (C) Reference spatial bird distribution pattern considering the hypothetical scenario without OWF. (D–F) Spatial bird densities with different OWF scenarios. (G–I) Local relative bird density changes due to different OWF scenarios, compared with hypothetical undisturbed scenario. For better visualisation, bird density changes (G–I) were rescaled separately for each plot and separately between −1 and 0 as well as 0 and 1 (i.e., with respect to increase (green) vs. decrease (red)), and the decrease and increase strengths are thus not directly comparable. In contrast, the colour scaling in (C–F) is directly comparable. All OWF scenarios later than 2019 are fictitious (but are based on currently discussed/planned scenarios in the German EEZ).

This spatial prediction data frame was subsequently multiplied by and assigned with the different years considered in this study, from 2003 to 2020, in a first step by merging the yearly spatial data with corresponding spatio‐temporal information from all the above considered covariates/predictors. For future predictions, 10 additional ‘virtual’ years were then augmented in a similar way, using the values from 2019 for all covariates, except the two OWF‐related variables. To investigate the impact of planned OWF extensions in German North Sea waters, we first simulated the implementation of ‘priority areas’ (grey‐shaded areas in Figure 2B) in virtual years 1–5 (including the corresponding calculation of the variables dist_OWF and sim_OWF), and the ‘reserved areas’ (grey‐striped areas in Figure 2B) were similarly integrated for the subsequent 5 virtual years. Notably, the exact number and time points of years used here were rather arbitrary, with minor impact on the predictions, given that predicted/simulated bird patterns appeared to stabilise quickly after each virtual OWF extension (cf., Figure 4A–B).

The final sdGAMM (as derived above) was used as a basis to predict bird densities in real and hypothetical scenarios. In particular, the estimation of anthropogenically induced changes was mainly based on comparisons between the (past, present or future) disturbed scenario (with OWF) versus the hypothetical scenario in which no OWF were constructed. These two scenarios were predicted for each year separately and related to each other as follows: first, the situation comprising existing, present or future OWF was predicted, using similar predictor variables to those used during the original fit of the model (possibly augmented by planned OWF data if future scenarios were considered—as described above). Second, the same data were used setting the variable dist_OWF constant to 20 km (representing only areas outside the local‐scale impact of OWF) and also setting sim_OWF to its initial constant value (i.e., no OWF driving large‐scale spatial redistribution of birds)—thus, the hypothetical scenario without OWF disturbance is considered.

As described above, the local effects of OWF on bird distribution were captured by the variable dist_OWF, which measures the distance to the closest operating OWF. However, estimates of the local dist_OWF effect can be confounded by baseline differences if bird densities in areas with future OWF already differed systematically from other areas before construction, either by chance or due to spatial co‐location with environmental gradients such as water depth or distance to the coast. In previous analyses for common murres (e.g., Peschko et al. 2024), effect estimates derived from BACI‐type analyses tended to indicate stronger local avoidance than estimates from non‐BACI analyses (e.g., Szostek et al. 2024), suggesting that non‐BACI specifications may underestimate the magnitude of local avoidance when baseline differences are not fully accounted for. As noted above, however, a classical BACI‐analyis is not possible within the present spatio‐temporal framework because it requires the data to be separated into two distinct ‘before’ vs. ‘after’ phases, which is not possible in a temporally consistent way, given that operation phases differ among different OWF clusters (Garthe et al. 2023; Peschko et al. 2024). To adjust the local‐scale OWF effect for potential baseline differences in a pragmatic way, we therefore applied a separate BACI‐based correction step. We first restructured the data according to the BACI‐design, as applied in Peschko et al. (2024) and fitted the above sdGAMM (including, if selected, the variable sim_OWF) but replacing the predictor $s\left(\text{dist}\_\mathrm{OWF}\right)$ by the expression OWF+PERIOD+OWF:PERIOD, where OWF is an indicator variable for birds in the 20 km surrounding of existing or future OWF, and the interaction term OWF:PERIOD was used to estimate the BACI‐consistent OWF‐induced reduction effect (for details on this approach see e.g., Garthe et al. 2023). This modified sdGAMM was termed baciGAMM, and the resulting BACI‐effect size was compared by the overall reduction induced by the $s\left(\text{dist}\_\mathrm{OWF}\right)$ in our regular sdGAMM in the 20 km surrounding of existing OWF, and a correction factor was finally derived and applied by the quotient of these two measures.

Although the PDE‐based redistribution step is formulated without intrinsic source or sink terms (i.e., conservative in the interior), our implementation uses an open rectangular simulation domain with Dirichlet boundary conditions (cf. above) and the redistribution index is subsequently combined with habitat preferences in the regression model. As a result, sim_OWF‐induced changes can lead to small deviations from strict number conservation when totals are compared over the German EEZ plus buffer. To account for this, we introduced an additional correction step. Here, disturbed and undisturbed scenarios were predicted separately for each year, while setting dist_OWF to 20km everywhere for both predictions (because dist_OWF induces legitimate local changes in expected bird numbers that should not enter the calibration factor). Subsequently, predictions of the undisturbed scenario were multiplied by the factor $N_{\text{disturbed}}/N_{\text{undisturbed}}$ (where N denotes the predicted total bird number in the considered area), which corrects for small number‐conservation deviations arising from large‐scale redistribution. In a second step, the disturbed scenario was re‐predicted using the actual dist_OWF values associated with existing or future OWF. This procedure re‐weights the sim_OWF‐induced spatial redistribution by local habitat preferences, yielding a pragmatic approximation of combined large‐scale redistribution and local habitat selection without increasing mechanistic complexity.

An additional correction step was applied to limit artefacts arising from extrapolation outside the empirically supported study area, which may present a potential problem during regression‐based approaches (cf. Introduction section): for each year and prediction separately, maximal predicted bird densities outside the study area (where empirical data were sparse) were locally cropped to the maximal predicted density value inside the study area. In particular, this inhibited unrealistically high predicted densities due to extrapolations outside the empirically assessed geographical range.

Yearly OWF‐driven relative changes in bird numbers within the entire study area were finally calculated by dividing the predicted numbers for the disturbed scenario by those of the (hypothetical) undisturbed scenario. Confidence intervals (95%) of these yearly values were calculated based on appropriate quantiles from 2000 resamples from the posterior distribution of the parameters of the final sdGAMM, in conjunction with the above predictions, that is, based on the variance–covariance matrix of model parameters (Wood 2017).

To identify areas with particularly strong declines or increases in bird density due to OWF, from the spatial distribution pattern of the disturbed scenario (considering a specific year) we pointwise subtracted the corresponding values related to the undisturbed scenario. Hence, local absolute, rather than relative changes were evaluated, with local negative values indicating a local OWF‐driven decline and positive values indicating an increase. Local decreases were generally stronger and/or more frequent than increases in the considered examples, and positive and negative values were thus rescaled separately between 0 and 1 (respectively −1 and 0) for each considered scenario, to improve visualisation. Hence, in the corresponding subfigures, the intensities of decreases (red) vs. increases (green) are not quantitatively comparable.

To assess the predictive robustness of alternative model formulations and to evaluate the contribution of different OWF‐related components beyond in‐sample fit, we conducted a time‐blocked cross‐validation. The full data set was partitioned into six non‐overlapping multi‐year blocks (each spanning approximately 3 years), which were withheld one at a time from model fitting and used exclusively for out‐of‐sample evaluation. This design preserves the temporal dependence structure of the data and avoids information leakage between training and test sets. Within each cross‐validation iteration, four candidate models were fitted to the training data: (i) a baseline model without any OWF‐related terms (M0), (ii) a purely distance‐based model including only the local distance‐to‐OWF effect $s\left(\text{dist}\_\mathrm{OWF}\right)$ (M1), (iii) a purely mechanistic redistribution model including only the large‐scale redistribution predictor sim_OWF (M2), and (iv) the combined model including both components (M3). All models were otherwise identical in structure and covariates, ensuring a fair comparison. Predictive performance was quantified using out‐of‐sample deviance on the held‐out data. Differences in out‐of‐sample deviance were calculated relative to the baseline model (M0), with negative values indicating improved predictive performance. Importantly, this cross‐validation analysis was not intended to establish causal superiority of any model formulation, but rather to assess the robustness and generalisability of the combined modelling framework under different development phases.

In summary, to account for natural temporal variability when comparing spatial patterns across years, the modelling framework thus explicitly separates OWF‐related effects from background temporal dynamics. Long‐term and non‐linear temporal variation unrelated to OWF was captured using a smooth function of year ($s\left(\text{YEAR}\right)$), complemented by autoregressive lag terms to account for temporal persistence at shorter time scales. In addition, decade‐specific random effects were included to absorb broader period‐level variation. local‐scale OWF effects were modelled via a distance‐dependent smooth that was calibrated using a BACI‐based correction, thereby reducing confounding with general temporal trends. Finally, scenario‐based predictions were constructed as contrasts under identical OWF‐independent conditions: from 2019 onwards, non‐OWF temporal effects were held constant, such that differences among scenarios and over time arise solely from changes in OWF configuration rather than from natural temporal variability.

### 2.5 Software

All statistical analyses, validation procedures and visualisations were performed using the statistical software R (R Core Team 2022). All PDE‐based analyses were based on the package *ReacTran* (Soetaert et al. 2010). Spatial analyses (among others of spatial autocorrelation) were carried out using the packages *sp* (Pebesma and Bivand 2005), *gstat* (Pebesma 2004), *rgdal* (Bivand, n.d.), geosphere (Hijmans and Karney 2021), *prevR* (Larmarange 2022) and *rgeos* (Bivand et al. 2017). *ggplot2* (Wickham 2009) was used for all other visualisations and plots. Finally, the package *mgcv* (Wood 2017) was used for GAMM analyses. All computations were performed using a desktop computer with eight physical cores (Intel Core i7–10700K CPU with 3.80 GHz). The computation time for all presented PDE‐ and GAMM‐based analyses (including resampling) was approximately 48 h (using parallel computing).

## 3 Results

The final selected sdGAMM retained both OWF‐related predictors ($s\left(\text{dist}\_\mathrm{OWF}\right)$ and sim_OWF), indicating that the data support both a local proximity response around OWFs and an additional large‐scale redistribution of murres across the wider system. The ecological relevance of these components is quantified below using scenario‐based temporal and spatial predictions (Figure 4); partial effect plots are given in Figure S3. For completeness, we summarise the corresponding model‐based effect magnitudes here. Although sim_OWF enters the regression model as a linear term, we want to point out that this does not imply a linear or directional movement response of animals. Instead, the linear coefficient scales a spatially complex, non‐linear redistribution pattern generated by the mechanistic simulation, such that the estimated effect reflects support for the large‐scale OWF‐specific redistribution structure rather than for example, a linear generic offshore displacement gradient. In particular, sim_OWF was associated with a strong increase in predicted occurrence, corresponding to a multiplicative effect of $\exp\left(3.60\right)\approx 36.5$ across the full range of the predictor. This pattern is consistent with substantial redistribution of murres across the wider study area beyond local OWF surroundings. In contrast, the distance‐based component was captured by a nonlinear smooth, indicating a pronounced, distance‐dependent reduction in occurrence in the vicinity of OWFs with recovery towards background levels at increasing distances (Figure S3). Indeed, our cross‐validation indicated that the combined model formulation (including both $s\left(\text{dist}\_\mathrm{OWF}\right)$ and sim_OWF) provided improved average out‐of‐sample performance relative to a baseline model without OWF‐related terms, and performed better on average than models relying on only one of the two OWF components (Figure S4).

Based on these relationships, we further evaluated and quantified the influence of existing and planned OWF on common murre population sizes and distribution in the German EEZ, using the predictions of the sdGAMM. Figure 4A shows the predicted relative change in the German North Sea population size (solid red line) compared with the hypothetical undisturbed scenario (blue line) depending on the year (*x*‐axis) for the different OWF scenarios (red numbered boxes). Notably, a slight (non‐significant) OWF‐induced increase in common murres in German waters was predicted until 2013. From 2014 onwards, however, predictions indicated a distinct decline in the German EEZ population under the OWF scenarios (Figure 4A), with each of the considered OWF extension scenarios causing a distinct step‐like decline. In terms of magnitude, the declines increase from the priority scenario to the priority plus reservation scenario, with the latter implying a reduction of the expected EEZ population to roughly one quarter of the hypothetical undisturbed level. The strongest decline was predicted for the scenario including reservation areas.

Based on the sdGAMM, we also estimated a nonlinear trend in common murre numbers summed up for the German EEZ (rescaled to an index of mean value 1.0), separately for the scenario with (red line) and without (blue line) existing and planned OWF (Figure 4B). Yearly numbers fluctuated strongly. Under the priority plus reservation scenario, predicted OWF‐related reductions extended below the range of variation observed in the time series (Figure 4B). Trends shown from 2020 onwards are scenario projections: for the undisturbed scenario, the summed abundance is held constant from 2020 onwards (because OWF‐independent influences cannot be reliably extrapolated), and the timing of the stepwise OWF expansion in the scenarios may not coincide with actual implementation.

The associated predicted spatial changes are given in detail in Figure 4C–I. Compared with the undisturbed scenario (Figure 4C), the situation in 2019 already showed a distinct displacement of birds, with higher predicted densities in more offshore regions (Figure 4D; also visible in the raw data in Figure S1A vs. S1B), and a (barely non‐significant) net reduction of 18.3% of the murre population in German waters (i.e., 81.7% remain). In particular, the south‐easterly (more nearshore) region, including OWF, showed large‐scale decreases in bird densities, whereas an increase was observed in the north‐western (far offshore) part of the EEZ (Figure 4D,G). This process was intensified by the addition of priority areas (total significant net reduction in the EEZ: 34.3%—Figure 4E,H—i.e., 65.7% remain), and in the case of additional OWF expansion within the reservation areas, with nearly the entire EEZ affected by significant losses and a predicted net reduction of 77.7% to only 22.3% of its size in the OWF‐undisturbed scenario (Figure 4F,I). These estimates represent scenario‐based net changes in summed abundance within the German EEZ and do not rely on perfect empirical separability of redistribution and loss components at the local scale. Under the latter scenario, predicted high‐density areas persisted mainly between wind‐farm clusters and at maximal distances to OWF.

## 4 Discussion

Throughout the following discussion, spatio‐temporal patterns are interpreted based on model‐based scenario contrasts in which natural temporal variability is explicitly accounted for, rather than on direct comparisons of raw spatial patterns across years. The observation that both OWF components ($s\left(\text{dist}\_\mathrm{OWF}\right)$ and sim_OWF) are retained and show consistent effects is in line with previous evidence for local‐ and large‐scale OWF responses of this species in the German North Sea (Peschko et al. 2024; Peschko, Mercker, and Garthe 2020). Although both OWF‐related predictors are highly significant, we focus the discussion on effect magnitudes rather than on model fit statistics, and assess these relationships in terms of their implied changes in predicted occurrence. In addition, cross‐validation (Figure S4) showed improved average out‐of‐sample performance for the combined formulation including both $s\left(\text{dist}\_\mathrm{OWF}\right)$ and sim_OWF, compared to models relying on only one OWF component.

A key implication of retaining both OWF components is that the response cannot be understood solely as a local footprint effect around OWFs: beyond near‐field avoidance, the results support a system‐scale redistribution that changes where displaced individuals are predicted to concentrate across the wider study area. This constitutes a shift from a purely footprint‐focused perspective towards an explicitly spatial perspective on redistribution and accumulation at larger scales, which is directly relevant when identifying where mitigation or protection measures would affect the highest expected densities of displaced individuals.

The redistribution component associated with sim_OWF shows a strong effect size (as reported in the Results), and its partial‐effect visualisation (Figure S3A) indicates a general large‐scale shift towards offshore waters with increasing OWF pressure, qualitatively consistent with the corresponding raw‐data plots (Figure S1). Importantly, the number‐conserving redistribution in the mechanistic simulations should not be interpreted as evidence for a closed population, but rather as a modelling device to disentangle spatial redistribution from concurrent changes in overall abundance within the focal area. The local‐scale effect is distance‐dependent: the partial effect of $s\left(\text{dist}\_\mathrm{OWF}\right)$ indicates a pronounced reduction in predicted occurrence in the immediate vicinity of operating OWF, with a gradual recovery towards background levels at increasing distances up to 20 km (Figure S3B). Accordingly, local‐scale effects are strongest close to OWF and attenuate with distance, whereas the sim_OWF component operates at the scale of the wider study area and is not confined to the local surroundings of OWF. Overall, results indicate two distinct components of the response to OWF: a large‐scale spatial redistribution and a local reduction in occurrence within the local‐scale surroundings of OWF.

Our qualitative and quantitative projections were within the orders of magnitude reported in Peschko et al. (2024), which focused on local‐scale effects only and did not include large‐scale redistribution. As a consequence, Peschko et al. (2024) did not describe the total loss of animals in the German EEZ, but rather the proportion that will suffer habitat loss around the OWF (local‐scale) according to a forecast based on animals being virtually ‘cut out’ based on measured displacement caused by operating OWF. In contrast, birds in the present study could also lose their habitat due to local‐scale effects, but our modelling approach could quantify those that disappeared completely from the study area vs. those that flew elsewhere inside the study area and were therefore not lost to the EEZ. Thus for the priority scenario, the current study predicted a 34.3% loss of animals that were present in the German EEZ (48% habitat loss in Peschko et al. 2024) because individuals that experienced habitat loss due to OWF in the closer surroundings ‘accumulated’ in areas without OWF influence thus did not leave the EEZ, whereas for the priority plus reservation area scenario, we predicted a 77.7% loss in the EEZ (69% habitat loss in Peschko et al. 2024). Thus, due to the large areas covered and influenced by OWF, very little space is left for murres inside the German EEZ without OWF influence. Hence a large part of the murres leave the German EEZ which leads to the higher numbers predicted in the current study.

The first‐mentioned difference between the percentages obtained by both approaches therefore reflects that, in 2019 and in the first future projection scenario, birds tend to accumulate in more offshore regions of the German EEZ. This spatial reorganisation partly dampens the effect of local‐scale habitat loss in the immediate surroundings of OWFs, because not all individuals are displaced from the EEZ; instead, some relocate to areas that maintain a sufficient distance to OWFs while still remaining within the EEZ. This should not be interpreted as an absence of negative effects, however, as such redistribution may still entail ecological costs, for example through reduced foraging conditions or increased stress due to local accumulation. This difference between the estimates of the two methods is reversed for the second expansion scenario including priority plus reservation areas, since here, the large‐scale redistribution (expressed by sim_OWF) adds to the losses on the local‐scale, as birds are now pushed beyond the EEZ‐boundaries because there are no longer enough undisturbed areas in the EEZ available at the spatial scales required by this species. Neglecting large‐scale redistribution can therefore lead to a different interpretation of regional‐scale consequences for abundance when such impacts are assessed based on habitat loss alone, depending on whether and how displaced birds accumulate within or outside the considered area. In particular, approaches that quantify the number of individuals experiencing local habitat loss capture an important but partial aspect of OWF impacts, without making statements about resulting changes in regional population size or about the ecological conditions encountered in alternative areas. When comparing the estimates of the present study with recent assessments of OWF‐related habitat loss (Peschko et al. 2024), however, various additional factors might result in additional deviations: Apart from the key difference that the present study augmented large‐scale redistribution to habitat loss (and in addition, EEZ population changes relative to the hypothetical undisturbed scenario have been quantified instead of estimates how many birds lose valuable habtat and thus have to search for suitable habitat eslewhere, without estimating were these animnals might move to), there were also various technical differences in the presented work vs. the study of Peschko et al. (2024): this includes a spatio‐temporal vs. a BACI‐approach, further differences with respect to data structure and evaluation, distinctly different spatial grid sizes (8 × 8 km vs. 2 × 2 km), different spatio‐temporal covariates, and a smooth dependency on the OWF‐distance vs. a corresponding binary variable. Any/all of these differences might influence the final estimates. Against this background, the local‐scale responses inferred in both studies are qualitatively consistent, in that both indicate a pronounced reduction in occurrence in the immediate surroundings of OWFs (cf. Figure S3 and below), while addressing different aspects of OWF‐related impacts. Rather than expecting identical quantitative estimates, the two approaches are complementary: one focuses on the number of individuals experiencing local habitat loss, whereas the present study additionally resolves how displaced individuals redistribute at larger spatial scales. When jointly interpreting results from both approaches, corresponding uncertainty ranges should therefore be considered as part of their respective inferential frameworks.

Taken together, our results and comparisons illustrate the key added inference enabled by explicitly accounting for large‐scale redistribution via sim_OWF. Approaches without sim_OWF quantify local avoidance or habitat loss around OWFs, which is an important aspect in its own right. However, if the question is whether and how the number of individuals present within a defined management area is expected to change following OWF construction, the joint consideration of $s\left(\text{dist}\_\mathrm{OWF}\right)$ and sim_OWF provides additional, policy‐relevant information, for example, by resolving whether displaced birds remain within the focal area or redistribute beyond its boundaries. In the present scenarios, the difference between the estimated losses reflects whether OWF‐induced redistribution primarily leads to accumulation in offshore parts of the EEZ or to displacement beyond the EEZ boundary, a distinction that cannot be inferred from distance‐based avoidance alone but requires the explicit representation of large‐scale redistribution captured by sim_OWF (cf. Figure S5).

From a conservation and management perspective, quantifying how many individuals experience local habitat loss around OWFs is highly relevant in its own right, because it indicates how many birds are forced to seek alternative habitat and are therefore affected by OWF development. At the same time, additional information on where displaced individuals relocate at larger spatial scales is important for spatial planning, for example to assess whether redistribution is expected to remain largely within the German EEZ or to occur predominantly beyond national boundaries. Importantly, redistribution or continued presence within the EEZ must not be interpreted as evidence that individuals encounter suitable habitat conditions there; potential ecological costs such as density‐dependent effects, altered foraging conditions, or increased stress can occur irrespective of whether birds remain inside the EEZ. Consistent with this complementary interpretation, the combined model formulation (including both OWF‐related terms) better reproduced the observed spatio‐temporal distribution of murres in cross‐validation (Figure S4). This reflects improved statistical prediction across years, rather than improved performance with respect to any single management or conservation endpoint. We emphasise that this does not imply universal superiority of one modelling approach over another; rather, dist_OWF and sim_OWF are designed to capture complementary processes at different spatial scales (local‐scale avoidance versus large‐scale redistribution), which have ecological implications that can and need to be considered independently. Their relative contribution depends on the ecological and spatial context.

When fitting the baciGAMM using different radii around OWF, the obtained orders of magnitude with respect to local‐scale OWF avoidance were similar to those presented in Peschko et al. (2024): for example, for a 1 km radius we measured a 91.4% reduction (Peschko et al. 2024: 91%), an 80.3% reduction for 5 km (Peschko et al. 2024: 80%), and a 72.9% reduction for a 10 km radius (Peschko et al. 2024: 76%). This demonstrates good robustness of these estimated local‐scale effects, despite the above methodological and technical differences between the studies. Interestingly, removing the variable sim_OWF from the baciGAMM (i.e., considering only the local‐scale effects) led to very similar orders of magnitude regarding the local‐scale effects (91.6%, 81.4% and 74.2% for 1 km, 5 km and 10 km radii, respectively). This demonstrates that both habitat losses (described by OWF or dist_OWF) and large‐scale redistribution (described by sim_OWF) captured two distinct processes acting at different spatial scales, given that local‐scale effects did not sensitively depend on the variable sim_OWF.

Our projections are likely conservative for several reasons. First, the current approach did not apply any constraints with respect to predicted local animal densities within the study area. In the considered example, this led to bird densities distinctly beyond those predicted for the undisturbed scenario (cf. Figure 4C vs. 4D,E) but it remains unknown if such high densities will occur in the future: Such high densities might either not occur because birds avoid very high densities e.g., due to inter‐ or intraspecific competition, or they may occur but lead to additional negative effects (such as increased stress/competition/fitness) not considered within our approach. Second, the present example only considered OWF, but other human pressures, including OWF‐associated ship traffic (Mendel et al. 2019) will increase simultaneously, further adding to the direct impact. In addition, cargo ship activity and OWF related ship traffic will increase in those areas not occupied by OWF, where our predicted remaining bird densities are highest. Third, planning of OWF areas in the German North Sea is currently ongoing, and the recent draft spatial development plan outlines even larger areas for OWF expansion, especially in the priority and reserved areas (BSH 2025). Fourth, our predictions assumed that areas outside the German EEZ could be used unreservedly by birds as evasive areas; however, adjacent countries may have similar OWF extension plans that might restrict evasion and thus make our prediction with respect to bird accumulations in the surroundings of the German EEZ too optimistic.

The predicted declines in abundance of common murres as a result of planned OWF expansion in the German North Sea are substantial. Especially in combination with the above reasons leading to an underestimation of the effects, it is plausible that this species may be reduced to very low numbers within German waters if OWF expansion is also realised in the reservation areas.

While the present analysis focuses on providing the best possible projections for common murres in the German EEZ, it is important to distinguish between the generality of the modelling framework and the transferability of quantitative effect sizes. The combined regression–mechanistic framework is, in principle, applicable to other species and regions. However, quantitative effect magnitudes are expected to be context‐dependent and species‐specific, and they may also depend on study design, spatial scale and season. Methodologically, reported differences in effect strength across studies may partly arise from differences in inferential design. In particular, several studies rely on non‐BACI or purely spatial designs, which do not explicitly account for pre‐construction differences in bird densities between future OWF sites and reference areas. As a consequence, estimated local avoidance effects may be biased if baseline spatial associations between birds and OWF locations are not corrected for. The BACI‐based calibration applied here aims to reduce this source of systematic confounding when estimating local‐scale avoidance effects, and the present framework further separates large‐scale redistribution from local avoidance. Against this background, studies from other regions of the North Sea and adjacent waters generally report distinct negative responses of common murres to operating offshore wind farms, but often with weaker average effect sizes than those estimated for the German EEZ (e.g., Vallejo Gillian et al. 2017; Trinder et al. 2024; Grundlehner et al. 2025). Importantly, these comparisons are not one‐to‐one: previous work often differs in spatial resolution and extent, and in some cases also in season (e.g., winter‐focused analyses versus our autumn focus). Apparent regional differences may therefore reflect a combination of ecological context (e.g., OWF density and configuration, spatial constraints of the system) and methodological (e.g., BACI vs. non‐BACI) and seasonal differences, rather than indicating a universally stronger response in the German EEZ. In this sense, the present results represent a context‐specific estimate for this species under the conditions of a densely developed and spatially constrained system, rather than a generic prediction for common murres elsewhere.

## 5 Conclusions and Outlook

In summary, we present a new analysis framework to estimate and predict changes in animal distribution and numbers caused by increased human activities within a specific area of interest. In particular, the framework synergistically uses mechanistic‐ and regression‐based modelling approaches to describe animal redistributions, along with regional losses/gains of animals as a result of human activities. By directly comparing the predicted scenarios with and without human pressures, the present approach does not rely on the problematic interpretation of regional trends, which may be influenced by various intrinsic and extrinsic processes that are difficult to estimate. In addition, this approach can be used to not only understand previous and current human‐induced changes, but also to predict the impact of different hypothetical future scenarios. We emphasise, however, that the present application fixes the class of redistribution models and explores a broad but constrained parameter space rather than propagating full structural uncertainty across alternative model classes; extending the framework towards formal ensemble‐based or comparative benchmarking analyses represents an important avenue for future work. Time‐blocked cross‐validation further supports that the combined regression–mechanistic formulation can improve predictive robustness on average, while we emphasise that performance varies across development phases and does not imply universal superiority over alternative approaches. Consistent with this, dist_OWF and sim_OWF should be interpreted as scale‐specific and complementary terms (local‐scale avoidance vs. large‐scale redistribution) rather than competing predictors.

We exemplarily applied our approach to investigate the current and future impacts of intensified OWF implementation on the numbers and distribution of common murres during autumn within the German North Sea. We demonstrated that OWF can have a pronounced negative effect on this species by inducing both redistribution and loss from the study area. Throughout, reported reductions refer to net changes in predicted abundance within the German EEZ, not to demographic mortality; differences arise because individuals may either redistribute within the EEZ or move beyond its boundaries, depending on the spatial configuration of OWF. In particular, OWF constructed up to 2019 reduced common murre numbers in German waters by approximately one fifth. The addition of OWF priority areas (representing planned expansions in the German EEZ up to around 2030) increased this reduction to approximately one third. If OWF reservation areas are further augmented by optional areas for future expansion in the German EEZ up to 2045, the predicted reduction within the EEZ increases to roughly four fifths. These estimates are conservative for several reasons and indicate that, under full implementation of priority and reservation areas, the abundance of common murres within the German EEZ could decline to very low levels. At the same time, these projections are conditional on current behavioural responses; changes such as habituation could alter future outcomes.

The predictive capacity of the present approach may be used in the near future to estimate and compare the impacts of alternative strategies, for example, during the expansion of alternative energy technologies in previously less‐disturbed habitats. Optimising both spatial and temporal implementation choices will help us to minimise the human impact on wildlife, with the ultimate aim of preserving biodiversity while fighting climate change.

## Author Contributions

**Moritz Mercker:** conceptualization (lead), formal analysis (lead), investigation (equal), methodology (lead), software (lead), writing – original draft (lead), writing – review and editing (equal). **Verena Peschko:** conceptualization (equal), data curation (equal), investigation (equal), supervision (equal), validation (lead), writing – review and editing (equal). **Kai Borkenhagen:** data curation (equal), investigation (equal), validation (equal), writing – review and editing (equal). **Nele Markones:** data curation (equal), investigation (equal), validation (equal), writing – review and editing (equal). **Henriette Schwemmer:** data curation (equal), investigation (equal), validation (equal), writing – review and editing (equal). **Volker Dierschke:** data curation (equal), investigation (equal), supervision (equal), validation (equal), writing – review and editing (equal). **Stefan Garthe:** data curation (equal), funding acquisition (lead), project administration (lead), resources (lead), supervision (lead), writing – review and editing (equal).

## Funding

This work was supported by Bundesamt für Naturschutz (FKZ:3520860100).

## Conflicts of Interest

The authors declare no conflicts of interest.

## Supporting information


**Figure S1:** Spatio‐temporally pooled bird‐count data (logarithm of murre number per km^2^—only non‐zero counts are shown) from autumn before OWF construction (A) and after OWF construction (B). Black dots represent all (pooled) observations (zero and non‐zero), coloured dots and their size are related to non‐zero bird densities, purple areas in (A) indicate areas with future OWF (before construction) and red areas in (B) represent operating OWF. Notably, (A) and (B) do not present a temporally homogeneous scenario, because construction and operation phases may differ among OWF.
**Figure S2:** Examples of mechanistic simulations considering the influence of offshore wind farms (OWFs—red) within German North Sea waters in 2019 (black lines represent coastline and boundaries of the exclusive economic zone (EEZ)). (A, B) Spatial distribution of the variable $c\left(t;x\right)$ representing OWF locations (red) augmented by an isotropic long‐range effect with strength γ (beige/yellow/green colour range depicts $c\left(x;t\right)$), evaluated for two different values of γ. (C–E) Example simulations of animal redistribution (avoidance) based on the OWF‐related variable $c\left(t;x\right)$. Different strengths of animal attraction/avoidance scaled by β (subfigures below; colour range represents $n\left(x;t\right)$), leading to quantitatively and qualitatively different animal distribution patterns.
**Figure S3:** Partial effects of OWF‐related predictors derived from the sdGAMM. (A) Large‐scale redistribution pattern associated with the predictor sim_OWF, shown for the 2019 configuration of operating OWF in the German EEZ during autumn. The pattern illustrates the spatial structure of the redistribution component captured by the model and corresponds to a snapshot of the large‐scale response. (B) Partial effect of distance to the nearest operating OWF (not yet BACI‐corrected), modelled as a nonlinear smooth of $\log\left(1+\text{dist}\_\mathrm{OWF}\right)$ and shown as a multiplicative effect on the expected response. Effects are expressed relative to conditions at the maximum considered distance to OWF (20 km; reference value = 1). Shaded areas indicate 95% confidence intervals. Partial effects illustrate the structure and magnitude of modelled relationships and should be interpreted in conjunction with the scenario‐based predictions presented in Figure 4.
**Figure S4:** Time‐blocked cross‐validation results summarised as mean differences in out‐of‐sample deviance relative to a baseline model without OWF‐related terms (M0). Shown are three alternative model formulations: a purely distance‐based model (M1), a purely mechanistic redistribution model (M2), and the combined model including both local distance‐based and large‐scale redistribution terms (M3). Points indicate mean differences across cross‐validation blocks, with error bars representing ±1 standard error. Negative values indicate improved predictive performance relative to the baseline model. Variability across cross‐validation blocks reflects differences among development phases and indicates context‐dependent model performance rather than uniform dominance of any single formulation.

## Data Availability

The R code used for data processing, modelling, and analysis, along with selected intermediate modelling results, is available as Supporting Information S1 in this article. Specifically, the provided files include the final R script (_R_Skript.R), key model results (Final_Res_mod1.RDS, Final_Res_mod2.RDS), resampling‐based confidence intervals (Final_Variance.RDS) and results from the PDE‐based parameter search (PDE_results1.RDS, PDE_results2.RDS). Due to legal and ethical restrictions, the original raw data cannot be shared, as they are subject to property rights and copyrights of the data owners (German Federal Agency for Nature Conservation). However, the provided code and intermediate modelling results allow for the reproduction of key analytical steps and methodological transparency.
