# Supplementary material for: Beyond Local Footprints: Disentangling Large‐Scale Redistribution and Local Abundance Responses to Offshore Wind Farms
Source: Ecol Evol. 2026 Mar 15;16(3):e73256. doi: 10.1002/ece3.73256 (PMC13093766; doi:10.1002/ece3.73256)
Supplement: Supplementary file 1 — Figure S1: Spatio‐temporally pooled bird‐count data (logarithm of murre number per km2—only non‐zero counts are shown) from autumn before OWF construction (A) and after OWF construction (B). Black dots represent all (pooled) observations (zero and non‐zero), coloured dots and their size are related to non‐zero bird densities, purple areas in (A) indicate areas with future OWF (before construction) and red areas in (B) represent operating OWF. Notably, (A) and (B) do not present a temporally homogeneous scenario, because construction and operation phases may differ among OWF. Figure S2: Examples of mechanistic simulations considering the influence of offshore wind farms (OWFs—red) within German North Sea waters in 2019 (black lines represent coastline and boundaries of the exclusive economic zone (EEZ)). (A, B) Spatial distribution of the variable ct;x representing OWF locations (red) augmented by an isotropic long‐range effect with strength γ (beige/yellow/green colour range depicts cx;t), evaluated for two different values of γ. (C–E) Example simulations of animal redistribution (avoidance) based on the OWF‐related variable ct;x. Different strengths of animal attraction/avoidance scaled by β (subfigures below; colour range represents nx;t), leading to quantitatively and qualitatively different animal distribution patterns. Figure S3: Partial effects of OWF‐related predictors derived from the sdGAMM. (A) Large‐scale redistribution pattern associated with the predictor sim_OWF, shown for the 2019 configuration of operating OWF in the German EEZ during autumn. The pattern illustrates the spatial structure of the redistribution component captured by the model and corresponds to a snapshot of the large‐scale response. (B) Partial effect of distance to the nearest operating OWF (not yet BACI‐corrected), modelled as a nonlinear smooth of log1+dist_OWF and shown as a multiplicative effect on the expected response. Effects are expressed relative to conditions at the maximum [file ECE3-16-e73256-s001.pdf]

1089 # Supporting information

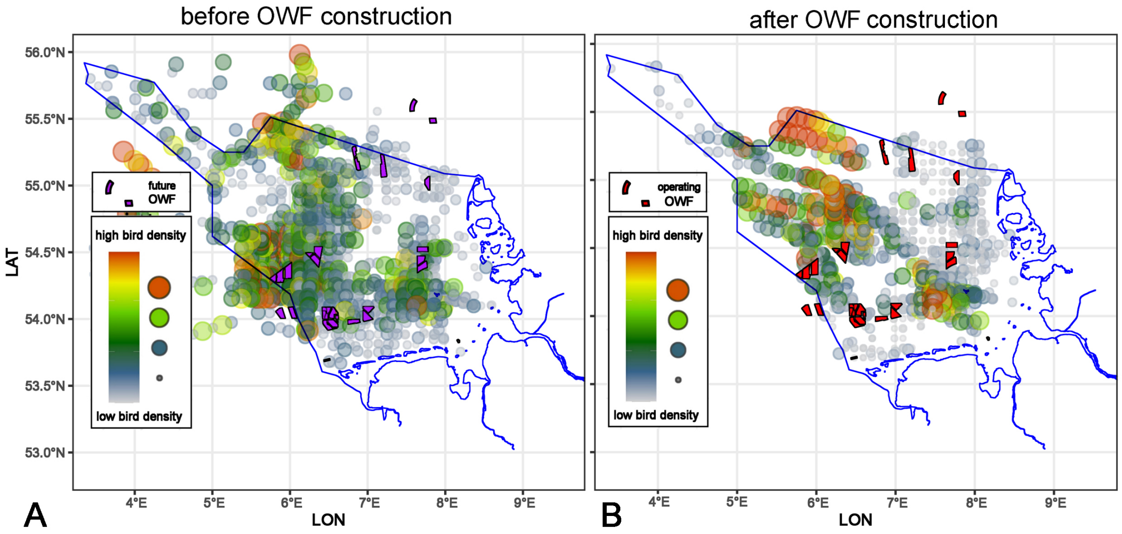

Figure S1. Spatio-temporally pooled bird-count data (logarithm of murre number per km^2 – only non-zero counts are shown) from autumn before OWF construction (A) and after OWF construction (B). Black dots represent all (pooled) observations (zero and non-zero), coloured dots and their size are related to non-zero bird densities, purple areas in (A) indicate areas with future OWF (before construction) and red areas in (B) represent operating OWF. Notably, (A) and (B) do not present a temporally homogeneous scenario, because construction and operation phases may differ among OWF.

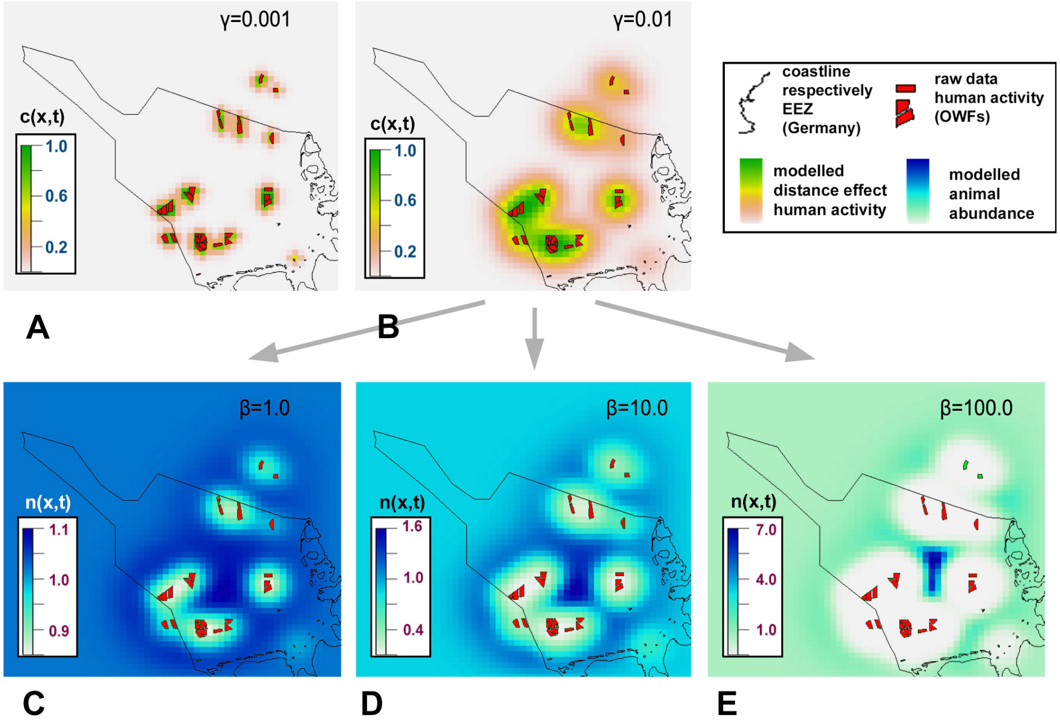

Figure S2. Examples of mechanistic simulations considering the influence of offshore wind farms (OWFs – red) within German North Sea waters in 2019 (black lines represent coastline and boundaries of the exclusive economic zone (EEZ)). (A)-(B) Spatial distribution of the variable $c(t;x)$ representing OWF locations (red) augmented by an isotropic long-range effect with strength γ (beige/yellow/green colour range depicts $c(x;t)$), evaluated for two different values of γ. (C)-(E) Example simulations of animal redistribution (avoidance) based on the OWF-related variable $c(t;x)$. Different strengths of animal attraction/avoidance scaled by β (subfigures below; colour range represents $n(x;t)$), leading to quantitatively and qualitatively different animal distribution patterns.

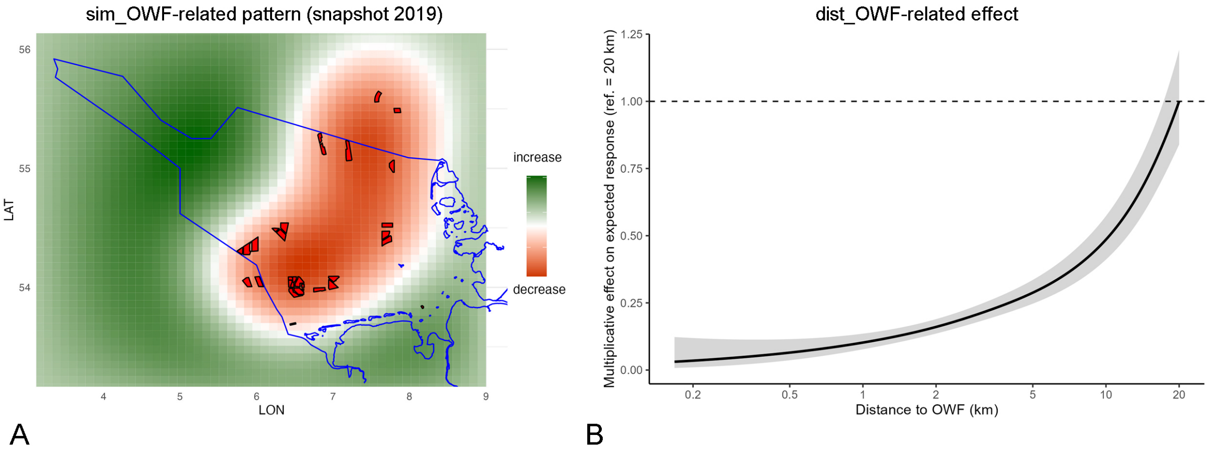

Figure S3. Partial effects of OWF-related predictors derived from the sdGAMM. (A) Large-scale redistribution pattern associated with the predictor sim_OWF, shown for the 2019 configuration of operating OWF in the German EEZ during autumn. The pattern illustrates the spatial structure of the redistribution component captured by the model and corresponds to a snapshot of the large-scale response. (B) Partial effect of distance to the nearest operating OWF (not yet BACI-corrected), modelled as a nonlinear smooth of $\log(1 + \text{dist\_OWF})$ and shown as a multiplicative effect on the expected response. Effects are expressed relative to conditions at the maximum considered distance to OWF (20 km; reference value = 1). Shaded areas indicate 95% confidence intervals. Partial effects illustrate the structure and magnitude of modelled relationships and should be interpreted in conjunction with the scenario-based predictions presented in Fig. 4.

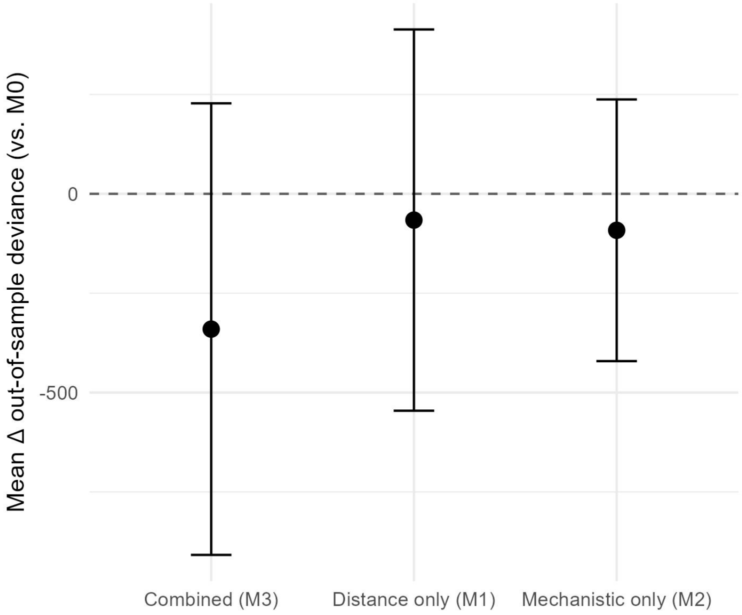

Figure S4. Time-blocked cross-validation results summarised as mean differences in out-of-sample deviance relative to a baseline model without OWF-related terms (M0). Shown are three alternative model formulations: a purely distance-based model (M1), a purely mechanistic redistribution model (M2), and the combined model including both local distance-based and large-scale redistribution terms (M3). Points indicate mean differences across cross-validation blocks, with error bars representing +/-1 standard error. Negative values indicate improved predictive performance relative to the baseline model. Variability across cross-validation blocks reflects differences among development phases and indicates context-dependent model performance rather than uniform dominance of any single formulation.
